# Bacterial diversity and biopotentials of Hamtah glacier cryoconites, Himalaya

**DOI:** 10.3389/fmicb.2024.1362678

**Published:** 2024-05-01

**Authors:** Purnima Singh, Shiv Mohan Singh, Takahiro Segawa, Prashant Kumar Singh

**Affiliations:** ^1^Indian Institute of Technology, Banaras Hindu University (IIT-BHU), Varanasi, India; ^2^Department of Botany, Banaras Hindu University, Varanasi, India; ^3^National Institute of Polar Research, Tachikawa-shi, Tokyo, Japan; ^4^Department of Biotechnology, Pachhunga University College, Mizoram University (A Central University), Aizawl, India

**Keywords:** cryoconites, glacier, Himalaya, bacterial diversity, culturable approach, metagenomics, enzymes, antibiotics

## Abstract

Cryoconite is a granular structure present on the glaciers and ice sheets found in polar regions including the Himalayas. It is composed of organic and inorganic matter which absorb solar radiations and reduce ice surface albedo, therefore impacting the melting and retreat of glaciers. Though climate warming has a serious impact on Himalayan glaciers, the biodiversity of sub-glacier ecosystems is poorly understood. Moreover, cryoconite holes are unique habitats for psychrophile biodiversity hotspots in the NW Himalayas, but unfortunately, studies on the microbial diversity of such habitats remain elusive. Therefore, the current study was designed to explore the bacterial diversity of the Hamtah Glacier Himalaya using both culturable and non-culturable approaches. The culturable bacterial count ranged from 2.0 × 10^3^ to 8.8 × 10^5^ colony-forming units (CFUs)/g at the different locations of the glacier. A total of 88 bacterial isolates were isolated using the culturable approach. Based on the 16S ribosomal RNA gene (16S rRNA), the identified species belong to seven genera, namely, *Cryobacterium, Duganella, Janthinobacterium, Pseudomonas, Peribacillus, Psychrobacter*, and *Sphingomonas*. In the non-culturable approach, high-throughput sequencing of 16S rRNA genes (using MiSeq) showed unique bacterial community profiles and represented 440 genera belonging to 20 phyla, namely, Proteobacteria, Actinobacteria, Firmicutes, Bacteroidetes, Chloroflexi, Acidobacteria, Planctomycetes, Cyanobacteria, Verrucomicrobia, Spirochaetes, Elusimicrobia, Armatimonadetes, Gemmatimonadetes, Deinococcus-Thermus, Nitrospirae, Chlamydiae, Chlorobi, Deferribacteres, Fusobacteria, Lentisphaerae, and others. High relative abundances of Proteobacteria, Actinobacteria, Firmicutes, and Bacteroidetes were observed in the samples. Phototrophic (Cyanobacteria and Chloroflexi) and nitrifier (Nitrospirae) in bacterial populations indicated sustenance of the micro-ecosystem in the oligotrophic glacier environment. The isolates varied in their phenotypic characteristics, enzyme activities, and antibiotic sensitivity. Furthermore, the fatty acid profiles of bacterial isolates indicate the predominance of branched fatty acids. Iso-, anteiso-, unsaturated and saturated fatty acids together constituted a major proportion of the total fatty acid composition. High cold-adapted enzyme activities such as lipase and cellulase expressed by *Cryobacterium arcticum* (KY783365) and protease and cellulase activities by *Pseudomonas sp*. strains (KY783373, KY783377-79, KY783382) provide evidence of the possible applications of these organisms. Additionally, antibiotic tests indicated that most isolates were sensitive to antibiotics. In conclusion, the present study contributed for the first time to bacterial diversity and biopotentials of cryoconites of Hamtah Glacier, Himalayas. Furthermore, the cold-adapted enzymes and polyunsaturated fatty acids (PUFAs) may provide an opportunity for biotechnology in the Himalayas. Inductively coupled plasma mass spectrometry (ICPMS) analyses showed the presence of several elements in cryoconites, providing a clue for the accelerating melting and retreating of the Hamtah glacier.

## Introduction

Ice masses store about 70% of freshwater, which sustains billions of people on planet Earth (Cook et al., 2016). Glaciers and ice sheets are the largest reservoirs of freshwater ecosystems, and climate change episodes are rapidly forcing the melting of these glaciers and ice sheets globally (Edwards et al., 2013). Understanding the biogeochemical processes at these places is crucial for predicting future changes, their effects on surrounding habitats, and potential losses of functional biodiversity. Cryoconites are dark-colored quasi-spherical granules found on glaciers and ice sheets across the globes, including the Arctic, Antarctic, and Himalayas (Kohshima et al., 1993; Hodson et al., 2008; Singh et al., 2017). The glacier's mass balance is greatly affected by the quantity and quality of cryoconite in a glacier (Nagatsuka et al., 2014). Cryoconites with a large area on the glacier surface primarily comprise organic and inorganic matter (Gerdel and Drouet, 1960; Hodson et al., 2008; Takeuchi et al., 2010; Singh et al., 2017).

Cryoconites are present on the surface or in quasi-cylindrical mini-depressions where the unique biome inhabits cryoconite holes (Wharton et al., 1985). Mineral dust inputs on glaciers depend on eolian dust deposition and transportation processes near the glaciers (Bøggild et al., 2010; Singh et al., 2013), which serve as nutrient sources for the microbes (Nagatsuka et al., 2014). Ice surface albedo is reduced by mineral dust (Bøggild et al., 2010), aggregation of cryoconite granules (Takeuchi et al., 2001; Hodson et al., 2008; Irvine-Fynn et al., 2012), microbial communities (Yallop et al., 2012; Singh et al., 2014), and meltwater production (Gruell, 2000). Due to the impact of climate change, warmer temperature transforms glacier zones into proglacial zones (Prowse et al., 2006), and subsequently, there is recolonization on glacier-retreated lands (Kaštovaká et al., 2005).

Abbot and Pierrehumbert (2010) opined the role of cryoconites as a potential melt catalyst in albedo-driven deglaciation of “Snowball Earth.” This unique habitat is a hotspot for culturable and non-culturable microbial diversity of Antarctica (Christner et al., 2003; Boetius et al., 2015; Webster-Brown et al., 2015; Obbels et al., 2016; Sommers et al., 2018; Poniecka et al., 2020) and Arctic (Edwards et al., 2011; Singh and Singh, 2011; Singh et al., 2014, 2020; Lutz et al., 2016; Uetake et al., 2016). Additionally, the studies on ecological succession (Williams et al., 2013), geochemistry of melt waters (Fountain et al., 2004; Tranter et al., 2004; Hodson et al., 2008; Bagshaw et al., 2013), and nutrient cycling (Stibal et al., 2012) have also been conducted. Sommers et al. (2018) advocated that environmental factors (lithology of the nearby mountains, height, and geographic location) also play a crucial role in regulating the cryoconite bacterial populations. Whole-genome analyses of a cryoconite bacteria showed unique genes for stress tolerance, DNA repair, enzyme activity, PUFA, and PGPR genes from the Arctic (Singh et al., 2015). Though cryoconite holes are very dynamic freshwater micro-ecosystems and are home to a rich biological diversity, studies at the range of spatial scales and a holistic understanding of this unique habitat are still a secret and thrust area of investigation (Cook et al., 2016).

Cryoconite holes have immense significance in understanding the sub-glacier ecosystems in the Himalayas. Himalayas have about 54000 glaciers and cover a vast geographical area (~60,000 square kilometers). Due to climate warming Himalayan glaciers are melting and retreating substantially, and may destabilize domestic, agricultural and industrial resources of people in the future. Very little work has been conducted on Himalayan cryoconites. The albedo-reducing, optical properties and microbial communities of cryoconites have been characterized from Nepali Himalaya (Takeuchi et al., 2001, 2010; Singh et al., 2020). The studies on biology and biogeochemistry of Indian Himalayan cryoconites and their interactions with climate remain elusive and are thrust areas for research. Hamtah Glacier is a well-researched benchmark glacier for mass balance, glacier melt runoff, and glacier meteorological and debris cover studies (GSI, 2009). This glacier is prone to climate change and loses its mass significantly during summer (Shukla et al., 2015; Mandal et al., 2016). Recently, Dhume et al. (2022) characterized fungal communities of Hamtah glacier. Bacterial diversity and biopotentials of Hamtah glacier is a gap area, therefore present study was conducted.

The aim of the current study was first, to investigate culturable and non-culturable bacterial communities present in Hamtah glacier cryoconites. Secondly, to characterize the biopotentials (cold-adapted enzymes, fatty acids, antibiotic resistance, carbon source utilization and physiological features). Furthermore, to know the physical properties of cryoconites, the elemental composition was also analyzed.

## Materials and methods

### Sampling site

**Figure 1:**
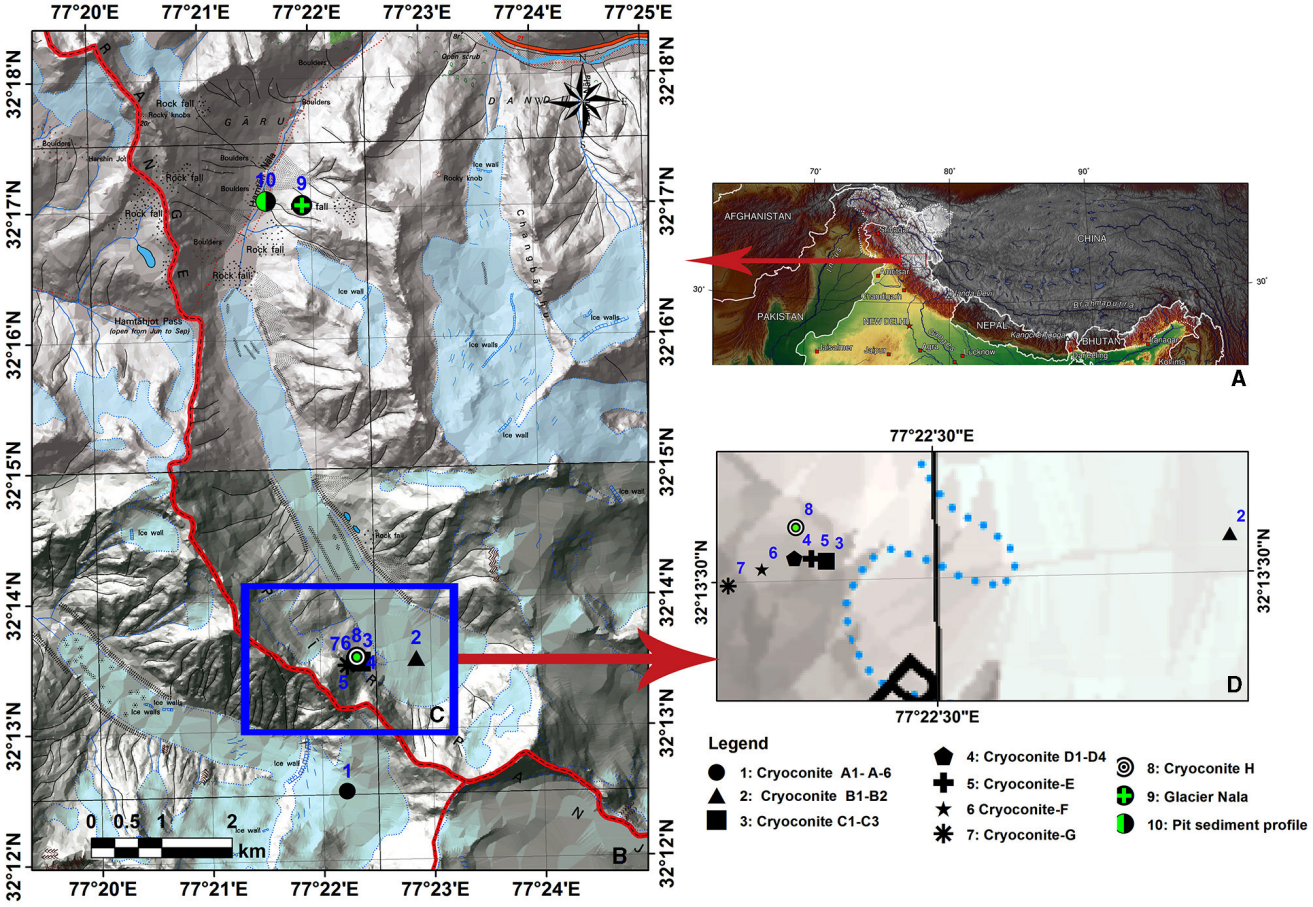
**(A)** Map showing Indian Himalayas, **(B)** Hamtah Glacier, **(C, D)** sampling locations.

Hamtah Glacier (32.24°N, 77.37°E) is an important glacier extending from south to north between 5000 and 4020 masl and covering an area of ~3 km^2^ and is ~6 km long (GSI, 2009). This glacier is located in the Chandra Basin on the northern slopes of the Pir-Panjal range of the Indian Himalayas. The landscape of the Hamtah Glacier and pictures of the sampled hole are shown in Supplementary Figure 1. Cryoconite samples were collected from seven different locations of Hamtah Glacier (Figure 1) using a sterile syringe and ampules. Before sampling, the pH and temperature of cryoconite holes were recorded. The temperature varied from 1.2 to 2.1°C, and pH ranged from 7.5 to 8.4 at different locations of the glacier. All the samples were stored at sub-zero temperatures and transported to the laboratory for further analysis.

### Isolation and culturing of bacteria

One gram of cryoconite was suspended in 9 mL saline solution and diluted serially (10^−1^, 10^−2^, and 10^−3^). To maximize the recovery of isolates, several bacteriological media (HiMedia, India) of different strengths (concentrations) were used in the present study. Enumeration of culturable bacteria was done using the spread plate method (0.1 ml) on Nutrient Agar (NA per liter: Beef powder 3 g, Sodium Chloride 8 g, Peptone 5 g and 20g Agar, pH 7.0), 1/10 NA, Marine Broth (MB per liter: Zobell Marine broth 20.25 g and 20 g agar, pH 7.6), 1/10MB, Antarctic Bacterial Medium (ABM per liter: 5 g peptone, 2 g yeast extract, and 15 g agar, pH 7.0), 1/10 ABM and R2Agar (R2Agar-HiMedia 18.12 g, pH 7.2) incubated at two different temperature 4°C and 15°C for 2–4 weeks for optimum growth of psychrophiles (Singh et al., 2016). To observe the emergence of bacterial colonies in a natural environment and compare them with laboratory incubators, the representative samples of each location (a total of 14 cryoconite samples) were also aseptically spread on readymade culture plates and incubated at the natural temperature of the Himalayas itself. The colony-forming units (CFUs) appearing after incubation were counted, and the CFU number per gm soil was calculated. Isolates for further study were picked based on colony morphology (outline, texture, color, etc.) from each plate. The pure bacterial isolates obtained through streaking techniques were preserved in cryovials with glycerol stock (70%) and finally stored at −80°C deep freezer. The unique distinctive cultures were submitted to the Microbial Culture Collection (MCC), Pune, India.

### Phenotypic characterization of the bacterial strains

Colony features were observed by a stereo-zoom microscope (Nikon SMZ1500). Carbon utilization tests were carried out using HiCarbo^TM^ kits (KB009, HiMedia, India) and incubated for 2–7 days at 4 and 15°C. ONPG (Ortho-Nitrophenyl-β-D-galactopyranoside) was used to detect β-galactosidase activity. Esculin, citrate, and malonate utilization tests were carried out to detect the capability of bacterial strains to utilize Esculin, citrate and sodium malonate as a sole carbon source. Growth experiments were performed on ABM plates at a range of temperatures (4, 15, 22, and 30°C), pH (3, 5, 7, 9, and 11) and salt concentrations (0, 2, 5, 7, and 15%). The unique bacterial isolates were screened for cold-active enzymes such as amylase, cellulase, lipase, and protease following standard protocol (Singh et al., 2014).

### DNA extraction, polymerase chain reaction, sequencing of culturable bacteria and phylogenetic tree construction

Genomic DNA was extracted following the standard method (Sambrook et al., 1989). 16S rRNA universal primers 16F27 (5′-CCA GAG TTT GAT CMT GGC TCA G-3′) and 16R1492 (5′-TAC GGY TAC CTT GTT ACG ACT T-3′) were used for PCR amplification. The amplified 16S rRNA gene PCR product was purified by PEG-NaCl precipitation. DNA sequencing was carried out using an automated DNA sequencer (ABI^®^ 3730XL, Applied Biosystems, Inc., Foster City, CA) at Microbial Culture Collection (MCC), NCCS at Pune, India. Essentially, sequencing was carried out from both ends using additional internal primers so that each position was read at least twice. Assembly was carried out using the Lasergene package followed by an EzTaxon search (Kim et al., 2012) for identification.

The nucleotide sequences of 47 bacterial isolates were deposited in the DNA data bank (NCBI) and assigned accession numbers are KY783365 to KY783401. Nucleotide sequences were subjected to NCBI BLAST search. Sequence alignment of the 16S rRNA region of each isolate was carried out using the Clustal W algorithm, Molecular Evolutionary Genetics Analysis (MEGA) 11.0 software was used for tree construction. The phylogenetic tree was constructed by neighbor-joining (NJ) method with 1000 bootstrap replicates of the phylogeny test, and partial deletion of data sub-set was used with a 95% cut-off setting in the partial deletion method. Red color phylogenetic trees indicate isolates/strains of the present study (sequences) and blue colored for closely related strains of the same species from the database.

### Non-culturable bacterial analyses: DNA extraction and high-throughput sequencing of 16s rRNA genes using MiSeq

Genomic DNA was extracted from 14 cryoconite samples (A, A2, A4, A5, A6, B, B2, C, D, D1, D2, D3, EHamtah, G, cryG) and one each from glacier Snout and glacier Nala/stream) using the FastDNA SPIN Kit (Soil DNA Extractionkit:MP Biomedicals, Santa Ana, CA). The bacterial community structure in the cryoconite samples was analyzed by sequencing the 16S rRNA gene using MiSeq sequencer (Illumina, San Diego, CA). Partial 16S rRNA gene sequences including the V3 and V4 regions were amplified. Bakt 341F and Bakt 805R primers with Illumina overhang adaptor sequences attached to their 5′ends were used. PCR amplification of the 16S rRNA gene, reaction clean-up, index PCR and sequencing were performed following Illumina methods for 16S rRNA gene sequencing library preparation, Pyrosequencing, Phylogenetic analyses and Community comparisons were done following standard procedure (Edgar et al., 2011; Segawa et al., 2014; Uetake et al., 2016).

### Fatty acid methyl ester analysis

The cultures were grown on a standard media Tryptic Soy Broth Agar (TSBA, Himedia) for FAME analyses, and also on respective isolate-isolation media at two standard psychrophilic temperatures 4^0^C and 15^0^C for 2 to 3 weeks. Three replicates of individual bacterial isolates were made by streaking on similar media plates and incubating them in similar conditions. The extraction and analysis of cellular fatty acid methyl esters (FAME) was carried out following standard protocol (Buyer, 2008). FAMEs analyses were carried out using a gas chromatograph (Agilent Technologies, CA, USA, Model 7890A), equipped with a flame ionization detector (FID), auto-sampler and capillary column (HP-5 column, 25 m × 0.2 mm × 0.33 μm). Hydrogen was used as carrier gas. The gas chromatography system was calibrated twice with a calibration standard no. 1300AA (MIDI, USA) before analyses of bacterial samples. Additionally, before running the cryoconite bacterial samples, the extraction efficiency of the protocol and authenticity of fatty acid peaks were verified by using a known fatty acid profile of a type strain (*Stenotrophomonas maltophili a* ATCC 13637^T^) as a positive control. The individual fatty acid peaks were identified based on the retention time of the standard run. The standard software and database were used (RTSBA6 of MIS, MIDI Inc., Newark, DE, USA) for analysis.

### Antibiotic susceptibility

The susceptibility of the bacterial isolates to 45 antibiotics (Supplementary Table 7) was determined by using the impregnated disc method (Bauer et al., 1966). The bacterial isolates were grown at 15°C in nutrient broth up to a turbidity of 0.08–0.13, optical density measured at 620 nm. The bacterial suspensions (100 μl) were spread onto the surface of the Mueller Hinton Agar medium plates, using sterile spreaders. The different antibiotic discs were placed over the individual bacterial lawns, and the plates were incubated at 15°C for 3–4 days. Petri dishes were observed for the formation of a zone of inhibition, if any. The zone formed was measured in millimeters.

### Elemental analyses of cryoconites

Cryoconite granules were finely grounded powdered samples were oven-dried at 110°C. An amount of 0.5 g of each sample was acid digested (3 mL of 69% sub-pure HNO_3_, 1 mL subpure 30% HCl and 1 mL of 30% H_2_O_2_) completely in the microwave at 180°C (Ethos 1, Milestone, Italy). The digested samples were analyzed using inductively coupled plasma mass spectrometry (ICP-MS), × Series II, Thermo Fisher Scientific, Bremen, Germany. CertiPUR ICPmulti-element standard (Merck, Darmstadt, Germany) was used for calibration. Elemental concentrations were measured in triplicate and were recorded in mg/kg as described previously (Singh et al., 2017).

## Results

### Characteristics of the glacier cryoconite samples

The pH and temperature in the cryoconite holes showed varied responses depending upon whether the holes are open or closed and, their sampling locations on the glacier. The water temperature in the cryoconite holes ranged between 1.2–2.1°C and pH 7.58–8.48. Cryoconites are brownish-black in color, organic carbon ranges from 0.57 to 1.12%, and total phosphorus 0.14 to 0.20 mg/g. The elemental composition showed the presence of 25 elements (Li, Be, Na, Mg, P, K, Ca, V, Cr, Mn, Fe, Co, Ni, Cu, Zn, As, Rb, Sr, Cd, Cs, Ba, Tl, Pb, Bi and U) in all cryoconite samples to varying amount. K, Mg, Mn, Fe, Ca, P and Na were dominant elements in samples (Table 1 and Supplementary Table 1).

**Table 1:** Culturable bacteria isolated from Hamtah glacier cryoconites, Himalaya.

**Sample name**	**Sampling location**	**Isolate no**.	**Sequence deposition no**.	**Total sequence length**	**Closest similarity with NCBI nucleotide database (18.03.2023, ISD)**	**Nearest match % similarity**
Cryoconites A	32 12. 543 N 77 22.210 E Elevation: 4,551 m, Temperature: 1.2, pH:8.48	A_2_6	KY783365	1,473	*Cryobacterium arcticum* strain A52	94.87
		A2(2)	MF467871	1,396	*Janthinobacterium svalbardensis* strain KCOM 1326	99.79
		A_2_7	KY783366	1,453	*Janthinobacterium svalbardensis* strain KCOM 1326	97.70
		A_4_5	KY783367	1,465	*Janthinobacterium* sp. strain M1_12	96.55
		A_4_P_1_	MF467867	1,373	*Janthinobacterium* sp. strain SNU WT3	99.64
		A_4_P_4_	KY783369	1,445	*Janthinobacterium svalbardensis* strain 20BR4L12	98.60
		A_4_P_5_	KY783368	1,470	*Janthinobacterium* sp. Zh1N-4	97.10
		A_4_P_6_	KY783370	1,468	*Duganella phyllosphaerae* strain MILP3	96.95
		A_4_P_7_	MF467869	1,363	*Janthinobacterium* sp. RHLS1-2	99.71
Cryoconite B	32 13.541 N 77 22.861 E Elevation: 4,365 m, Temperature:1.4, pH:7.6-7.8	B_2_f	KY783371	1,457	*Janthinobacterium* sp. strain xPrg51	98.08
		B_2_(G)	MF467863	1,385	*Janthinobacterium* sp. strain BWHT3	99.78
		B_2_3	KY783372	1,458	*Janthinobacterium* sp. strain BSLA3	97.75
		B_2_6	KY783373	1,490	*Pseudomonas marginalis* strain HCR18v	92.12
		B_2_8	KY783374	1,476	*Janthinobacterium svalbardensis* strain 20BR4L12	94.79
		B_2_12	KY783375	1,445	*Janthinobacterium svalbardensis* strain 20BR4L12	97.66
		B2-14	MF467870	1,320	*Janthinobacterium svalbardensis* strain KCOM 1326	99.54
		B_2_P_1_	KY783376	1,460	*Janthinobacterium* sp. HLX1	96.97
		B_2_P_3_	KY783377	1,455	*Pseudomonas* sp. Ln4B.12g	97.33
		B_2_P_4_	KY783378	1,466	*Pseudomonas* sp.MS-A-S3	97.30
		B_2_P_6_	KY783379	1,458	*Pseudomonas extremaustralis* strain 20BR4AA9	98.49
		B2-P7	MF467873	1,350	*Sphingomonas* sp. 2PM11	99.39
		B_2_P_9_	KY783380	1,450	*Janthinobacterium* sp. strain BFS1HT1	97.80
		B_2_P_10_	KY783381	1,568	*Janthinobacterium svalbardensis* strain ACR1p	94.67
		B_2_P_11_	KY783382	1,450	*Pseudomonas extremaustralis* strain 20BR5VA1	97.99
		B_2_P_13_	KY783383	1,453	*Janthinobacterium lividum* strain KOPRI 25648	97.52
		B_2_P_14_	KY783384	1,587	*Janthinobacterium svalbardensis* strain A2(2)	90.73
		B_2_P_15_	KY783385	1,447	*Janthinobacterium lividum* strain MWHB2	97.80
Cryoconite-E	32 13.523 N 77 22.346 E Elevation: 4,365 m, Temperature:2.1, pH:8.04	EI_1_	KY783386	1,447	*Janthinobacterium svalbardensis* strain 20BR4R40	97.42
		EI_2_	KY783387	1,479	*Janthinobacterium svalbardensis* strain IHBB	95.95
		EI_3_	KY783388	1,468	*Janthinobacterium svalbardensis* strain NJ-XFW-1-C	97.62
		EI_4_	KY783389	1,465	*Janthinobacterium* sp. sgn 135	97.47
		EI_5_	KY783390	1,450	*Janthinobacterium* sp. strain M1_12	98.15
		EI_6_	KY783391	1,455	*Janthinobacterium* sp. strain BFS1HT1	97.45
		EI_8_	KY783392	1,477	*Janthinobacterium tructae* strain Y2PP14	94.93
		EI_9_	KY783393	1„446	*Janthinobacterium lividum* strain KOPRI 25720	97.99
		EI_10_	KY783394	1,483	*Janthinobacterium* sp.MS-A-S1	95.50
		EI-11	MF467865	609	*Janthinobacterium lividum* strain EIF2	99.02
		EI_13_	KY783395	1,460	*Pseudomonas* sp. strain S2INA_B3	97.43
		EI-16	MF467866	1,372	*Janthinobacterium svalbardensis* strain A4P1	99.20
		EI_17_	KY783396	1,448	*Janthinobacterium* sp. strain M1_12	97.70
		Ecry2	KY783397	1,476	*Psychrobacter alimentarius* strain JM48	96.82
		Ecry4	MF467864	1,418	*Bacillus simplex* strain ER20	99.50
		Ecry7	MF467868	1,376	*Janthinobacterium* sp. strain M1_12	99.49
Cryoconite-F	32 13.520 N 77 22.520 E Elevation: 4,550 m, Temperature: 2.1, pH:8.04	HF_5_	KY783398	1,447	*Psychrobacter alimentarius* strain JM48	98.11
		HF_6_	KY783399	1,478	*Pseudomonas* sp. strain KP-10-2	95.62
		HF_8_	KY783400	1,443	*Pseudomonas* sp. strain PCH61	98.91
		HF_9_	KY783401	1,467	*Pseudomonas* sp. strain FH1	97.30

### Bacterial count and characteristics of the isolated strains

The culturable bacterial count (colony forming units, CFUs) on various bacteriological media ranged from 2.0 × 10^3^ to 8.8 × 10^5^ CFUs per g. Of the numerous CFUs that appeared on the culture media plates, 47 distinct isolates were purified for further studies (Table 1). The culture colonies showed different colors (white, cream, yellow and orange), and morphological features (entire margin, opaque and smooth texture, and convex pulvinate to raised elevation). The shapes of bacterial cells were short to long rods. Most of the isolates showed growth temperature ranges from 4 to 22^o^C. The optimum temperature for the growth of culturable isolates was 15^o^C, therefore indicating psychrophilic. The isolates showed salt tolerance at 1 and 7% and optimum growth at pH 6.0 to 7.0. The 16S rRNA gene sequences of 47 bacterial isolates were deposited to GenBank (NCBI) with accession numbers (KY783365 to KY783401, MF467864 to MF467871, and MF467873) (Table 1).

### Carbon utilization ability of the isolated strains

Of the 35 carbon sources tested, most of the isolates showed varied responses for carbon utilization tests (Supplementary Table 2). Amongst the isolates tested, *Peribacillus frigoritolerans* E-Cry-4 utilized 25, *Cryobacterium arcticum*-A2–6 utilize 24, *Pseudomonas* simiae-B2–6 utilized 26, *Psychrobacter pulmonis* E-Cry-2 utilize 27, *Sphingomonas glacialis* B2-P7 utilize 17, *Janthinobacterium* svalbardensis B2–8 can utilize as much as 4 (ONPG, esculin, citrate and malonate), of the 35 carbon sources tested. Except isolate E-Cry-2 none of the isolates were capable of utilizing sorbose.

### Extracellular enzymatic activities

The 45 bacterial cultures that were screened for enzyme production showed varied responses. Four isolates were positive for amylase, 12 for cellulase, 14 for lipase, and 20 for protease (Supplementary Table 3). Temperature showed a vital role in enzyme production. Cellulase, lipase and protease production decreased with an increase in temperature from 4 to 22°C. In contrast, amylase production increased with an increase in temperature. *Cryobacterium arcticum*-A2–6 was the most promising culture as it has strong cellulase and lipase activity, while 4 other cultures (B2P3, B2P4, B2P6, B2P11) produced three different enzymes: cellulase, lipase and protease. Four cultures (B2–6, HF6, HF8, HF9) indicated strong activity for cellulase and protease. Three cultures (E-cry 4, HF6, HF8) showed amylase activity (Supplementary Table 3).

### Phylogenetic analyses/taxonomic analysis of culturable bacteria

**Figure 2:**
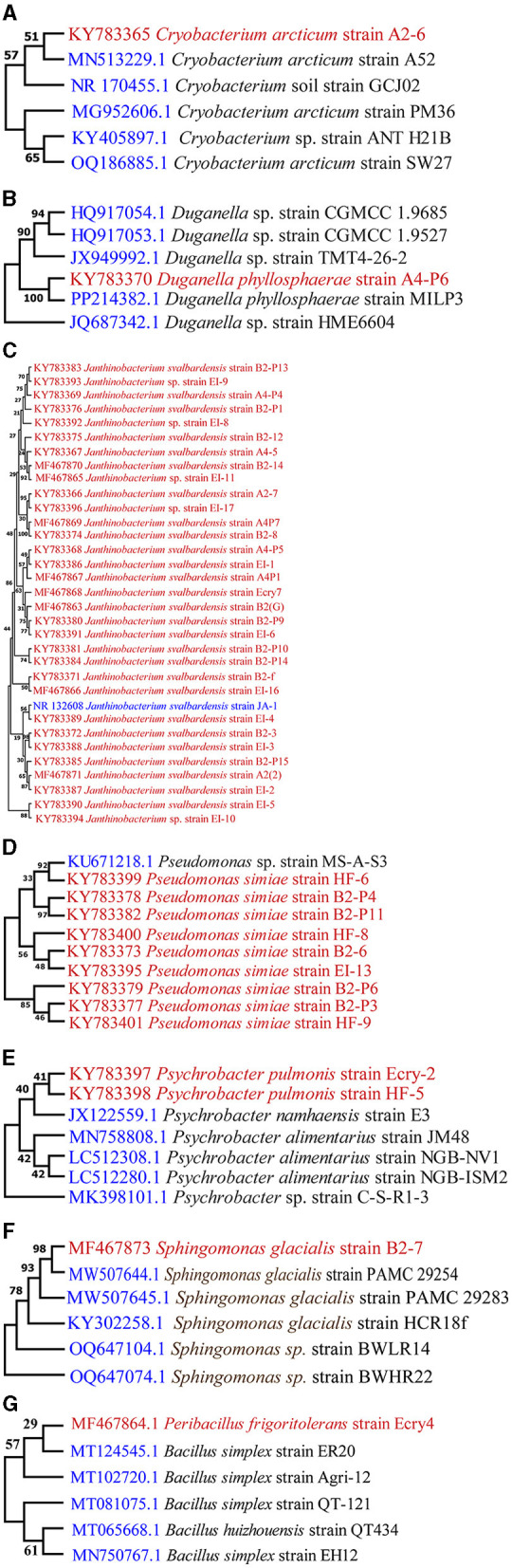
Phylogenetic trees of Culturable bacteria: **(A)** *Cryobacterium*, **(B)** *Duganella*, **(C)** *Janthinobacterium*, **(D)** *Pseudomonas*, **(E)** *Psychrobacter*, **(F)** *Sphingomonas*, **(G)** *Peribacillus*.

The total sequence lengths after alignment, the closest sequence similarities % with the database, and the NCBI sequence deposition numbers are given in Table 1. Sequence alignment was done using clustalW algorithm. The sequence analysis of the 16S rRNA gene domain of isolate *Peribacillus frigoritolerans* Ecry4 (MF467864) indicated their closest relationship to NCBI database species *Bacillus simplex* strain ER20 (99.50%), *Cryobacterium* A2–6 (KY783365) showed closest relationship with species of *Cryobacterium arcticum* strain A52(94.87%). *Duganella* A_4_P_6_ (KY783370) indicated *their closest relationship with the species of* *Duganella phyllosphaerae* MILP3 (96.95%). Sequence analysis of 32 bacterial isolates of *Janthinobacterium* **(**A2(2), A_2_7, A_4_5, A_4_P1, A_4_P_4_, A_4_P_5_, A_4_P_7_, B_2_f, B2(G), B_2_3, B_2_8, B_2_12, B2–14, B_2_P_1_, B_2_P_9_, B_2_P_10_, B_2_P_13_, B_2_P_14_, B_2_P_15_, EI_1_, EI_2_, EI_3_, EI_4_, EI_5_, EI_6_, EI_8_, EI_9_, EI_10_, EI_11_, EI_16_, EI_17_ and Ecry7) indicated their closest relationship to species of *Janthinobacterium svalbardensis* JA-1 (93.01 to 99.79%). Nine bacterial isolates of *Pseudomonas* (B_2_6, B_2_P_3_, B_2_P_4_, B_2_P_6_, B_2_P_11_, EI_13_, HF_6_, HF_8_, HF_9_) showed their closest relationship (92.12 to 98.91%) to the species of *Pseudomonas marginalis* strain HCR18v and *Pseudomonas extremaustralis* strain 20BR5VA1. Isolate *Sphingomonas glacialis* B2-P7 (MF467873) indicated their closest relationship with species of *Sphingomonas* sp. 2PM11 (99.39%). Sequence analysis of 2 bacterial isolates of *Psychrobacter pulmonis* Ecry2 (KY783397) and HF_5_ (KY783398) indicated their closest relationship with species of *Psychrobacter alimentarius* strain JM48 CECT 5989 (96.82) and *Psychrobacter namhaensis* strain E3 98.11%). The affiliation of the representative isolates of culturable bacteria is shown in a phylogenetic tree (Figures 2A–G).

The bacterial isolates from the cryoconites of different locations in Hamtah glacier belonged to seven different genera, namely, *Cryobacterium, Duganella, Janthinobacterium, Pseudomonas, Sphingomonas, Peribacillus* and *Psychrobacter* (Table 1). The number of species at each location varied from 2 to 4, while number of isolates varied from 4 to 18. The most predominant species was *Janthinobacterium svalbardensis* followed by *Pseudomonas* simiae. Genera *Cryobacterium, Duganella, Peribacillus* and *Sphingomonas* were represented the least. Glacier location E showed maximum culturable diversity, representing four different species, while location F of glacier showed minimal diversity, representing only two species.

### Analyses of non-culturable bacteria using MiSeq high-throughput sequenced data of 16S rRNA genes

**Figure 3:**
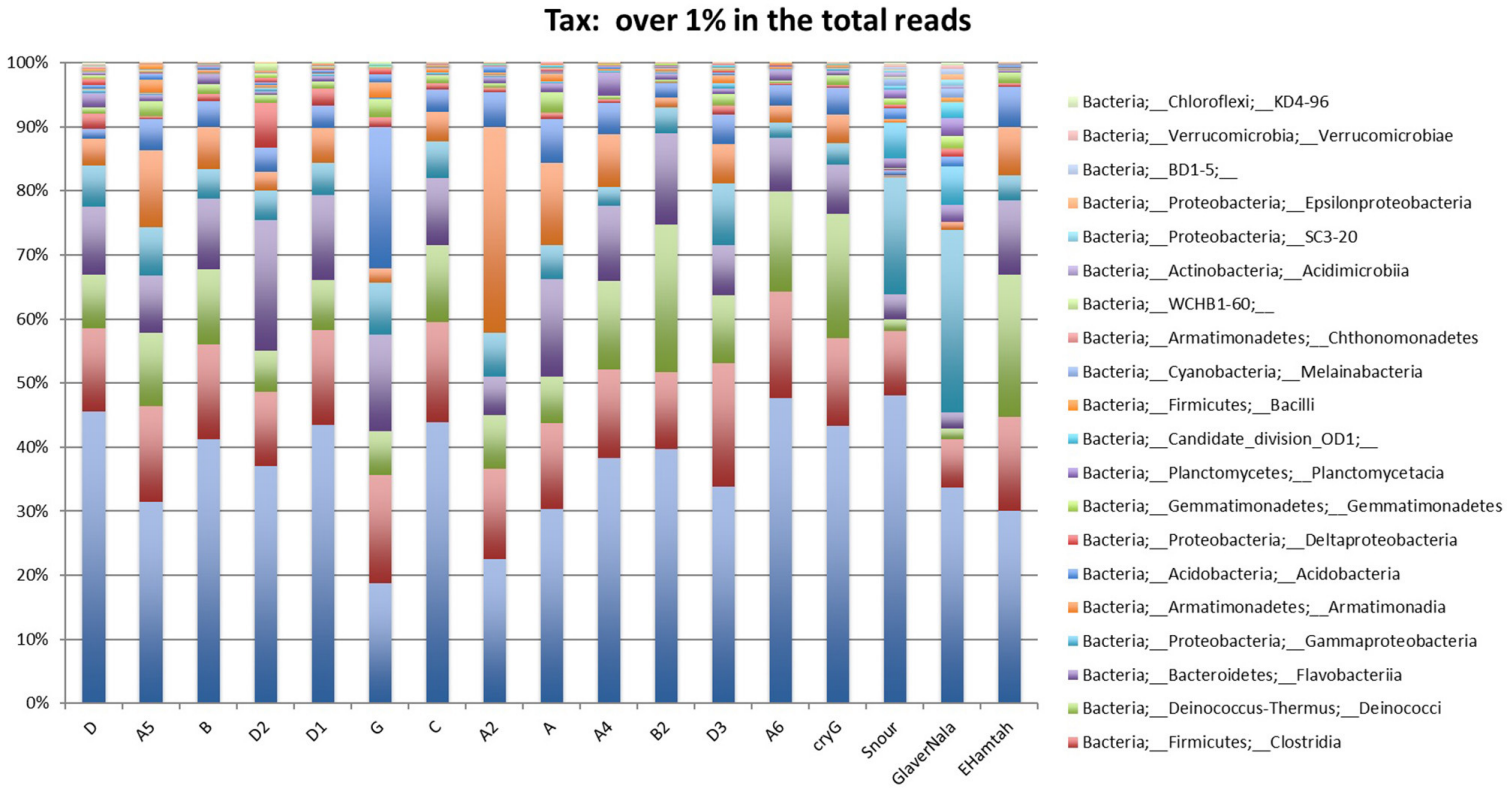
Non-culturable Bacteria: showing relative abundance (%) of bacterial phyla in Hamtah glacier cryoconites, snout, and stream (Nala).

**Figure 4:**
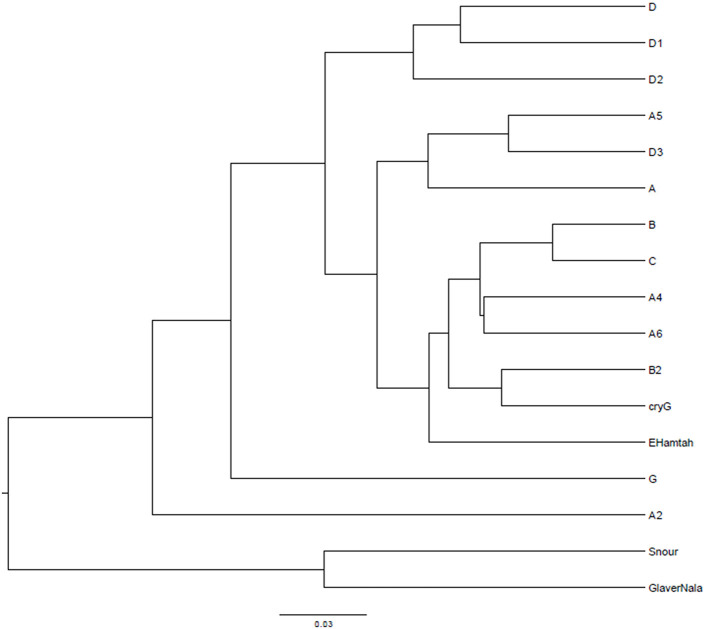
UniFrac dendrogram showing the similarity of bacterial community structures between sampling sites.

After curation of the MiSeq dataset, 16S rRNA gene sequences representing the non-culturable bacterial populations from the cryoconite samples across Hamtah glacier, Himalaya. The profiles represent eight major lineages: Proteobacteria was the most dominant (207 genera, 64 families), followed by Actinobacteria (80 genera, 28 families), Firmicutes (52 genera, 19 families), Bacteroidetes (51 genera, 12 families), Cyanobacteria (10 genera, two families) with other member phyla such as Chloroflexi (eight genera and seven families), Acidobacteria (five genera and three families), Planctomycetes (six genera, one family), Verrucomicrobia (five genera, four families) Deinococcus-Thermus (three genera, three families), Chlamydiae (three genera, two families), Spirochaetes (three genera, two families), Armatimonadetes (two families and two genera), The phyla Caldiserica, Deferribacteres, Elusimicrobia, Fusobacteria, Gemmatimonadetes, Lentisphaerae, and Nitrospirae represented least (one family and one genus in each phyla) along with others unknown taxa (Supplementary Table 4). In the present study, Proteobacteria is an important bacterial phylum and represent maximum number of bacteria belonging to five classes such as Betaproteobacteria (77 genera, 11 families), Alphaproteobacteria (54 genera, 19 families), Gammaproteobacteria (47 genera and 15 families), Deltaproteobacteria (23 genera and 16 families) and Epsilonproteobacteria (six genera and three families). Proteobacteria, Actinobacteria, Bacteroidetes and Firmicutes were well represented in all of the sampling sites. Armatimonadetes, Caldiserica, Deferribacteres, Elusimicrobia, Fusobacteria, Gemmatimonadetes, Lentisphaerae and Nitrospirae were present least in samples. The relative abundance (%) of bacterial phyla present at different sampling sites are shown in Figure 3 and Supplementary Table 5. The bacterial profile results indicate that taxa distribution exhibits a specific distribution pattern along the different glacier cryoconites, glacier snouts and glacier streams. The similarity of bacterial community structures between sampling sites is shown in a UniFrac dendrogram (Figure 4).

### FAME analysis

Six representative bacterial taxa were characterized for cellular fatty acid composition. *Janthinobacterium svalbardensis* B_2_8 (KY783374), Gram-negative, and Betaproteobacteria belong to the Oxalobacteraceae family. *Pseudomonas* simiae B_2_6 (KY783373) Gram-negative, Gammaproteobacteria, belonging to the family Pseudomonadaceae. *Psychrobacter pulmonis* Ecry2 (KY783397) Gram-negative, Gammaproteobacteria, belonging to the family? Moraxellaceae. *Peribacillus frigoritolerans* Ecry4 (MF467864) Gram-positive, Firmicutes, belonging to the family Bacillaceae. *Cryobacterium arcticum* A_2_6 (KY783365) Gram-positive bacterial strains belong to family Microbacteriaceae. *Sphingomonas glacialis* B_2_P-7 (MF467873) Gram-positive, Alphaproteobacteria, belonging to the family Sphingomonadaceae.

In the Hamtah glacier bacterial species, the branched fatty acids varied in composition from a minimum of 0.17% as in *Janthinobacterium svalbardensis* B_2_8, to a maximum of 96.59% as in *Cryobacterium arcticum* A_2_6. In *Janthinobacterium svalbardensis* B_2_8 the low % of branched fatty acids was compensated by a high proportion of unsaturated fatty acids (63.24%). The unsaturated fatty acid composition in all the bacterial species varied in composition from a minimum of 58.4% as in *Janthinobacterium svalbardensis* B_2_8, to a maximum of 90.72% as in *Peribacillus frigoritolerans* Ecry4 (Supplementary Table 6). The saturated fatty acid composition in all the bacterial species varied in composition from a minimum of 2.39% as in *Sphingomonas glacialis* B_2_P-7, to a maximum of 41.24% as in *Janthinobacterium* svalbardensis B_2_8 (Supplementary Table 6).

### Antibiotic resistance patterns of the isolated strains

Cryoconite bacterial isolates were subjected to 45 antibiotic screening tests. The result showed that most of the isolates were sensitive to antibiotics such as Amikacin, Carbenicillin, Cefoperazone, Ceftazidine, Chloramphenicol, Ciprofloxacin, Doxycycline hydrochloride, Gatifloxacin, Gentamicin, Kanamycin, Levofloxacin, Lomefloxacin, Meropenem, Nalidixic Acid, Netillin, Norfloxacin, Ofloxacin, Rifampicin, Streptomycin, Tetracycline, and Tobramycin (Supplementary Table 7). The sensitivity to other antibiotics tested varied from isolate to isolate. Clidamycin and Methicillin were the least potent antibiotics tested and were sensitive to only two isolates (Ecry-2 and Ecry-4). Of the isolates screened against 45 antibiotics, B2–8 and B2–6 were the most resistant, showing resistance to 19 and 21 antibiotics, respectively. Other isolates showed varied resistance profiles toward the antibiotics.

## Discussion

The main aim of the current study was to investigate the bacterial diversity, biopotentials and properties of cryoconites of Hamtah glacier, Himalaya. The pH of the cryoconite holes observed in the present study ranged from 7.5 to 8.4, resembling earlier data pH 5.9–9.6 from Canada glacier Antarctica (Mueller and Pollard, 2004), and pH 7.1–8.6 from Arctic glaciers (Singh et al., 2014). The pH properties of cryoconites holes depends on mineral contents, microbial activities and glacier environment in which it is found (Tranter et al., 2004). The organic carbon content in the present study was low (0.57%−1.12%) confirming the oligotrophic nature of cryoconite habitat. The similar data have also been reported from Arctic (1.07%−2.5%) by Singh et al. (2014), and 5.4%−9.9% from Nepali Himalaya (Takeuchi et al., 2001). Of 25 elements observed in cryoconites of the present study, Mg, Fe, Mn, Ca, P, and Na were dominant, as observed in previous studies (Singh and Singh, 2011; Singh et al., 2017). The accumulation and release of these inorganic material may impact supraglacier and proglacier ecosystems of Himalayas. The elemental content is mainly input from atmospheric, hydrologic and mineral resources, and this finding is further supported by Mueller et al. (2001) and Singh et al. (2017). The bacterial CFUs that emerged on the media plates ranged from 2.0 × 10^3^ to 8.8 × 10^5^ CFUs per g at the different locations of Hamtah glacier studied. The bacterial CFU data similar to the current observation are also reported from Arctic cryoconites (2.7 × 10^3^ to 8.8 × 10^4^ per g, Singh et al., 2014) and from Antarctic cryoconite melt water (8.8 × 10^4^ per ml, Christner et al., 2003).

The bacterial isolates could grow at 4 to 22°C, except for one at 30°C. The optimum temperature of growth was 15°C. The Hamtah glacier isolates profusely grow at 4 and 15°C as compared to 22°C resembles with previous studies from Antarctic and Arctic (Christner et al., 2003; Singh et al., 2014). Although the culturable bacterial diversity in Hamtah cryoconites is limited, but their strains differ widely in their antibiotic sensitivity characteristics. In the present study, the sensitivity of isolates toward antibiotics varied, as recorded in earlier studies from Arctic cryoconites (Singh et al., 2014). The most of the isolates indicated low resistance toward antibiotics in Himalaya similar to Polar regions. Segawa et al. (2012) reported that the Antarctic region has the least antibiotic resistance genes, followed by the Arctic region. The organic carbon sources are important for microbial metabolism but it limited in oligotrophic environment of cryoconite holes. The carbon source utilization tests showed that bacterial isolates from the Himalayan cryoconites prefer monosaccharides similar to Arctic cryoconite (Singh et al., 2014). The limited available organic and inorganic matter in cryoconite holes has also been reported from Antarctica (Foreman et al., 2007). Psychrophilic organisms can produce cold-active enzymes which have immense applications in Agriculture, health and industry (Feller and Gerday, 2003). The 45 bacterial isolates of Hamtah glacier cryoconites showed activities for one or more enzymes (amylase, cellulase, lipase, protease) either at 4°C, 15°C, and (or) 22°C. The ability of these bacterial isolates to produce different enzymes speaks about their biopotentials in sub-glacial environments. Some of the isolates of the present study showed strong cellulase, lipase and protease activity, similar to Arctic cryoconites (Singh et al., 2014). Additionally, there are reports of extracellular enzyme activities by Arctic bacteria (Reddy et al., 2009; Yu et al., 2009) and Himalayan fungi (Dhume et al., 2019).

The taxonomic analyses of bacteria using a culturable approach showed the presence of seven genera *Cryobacterium, Duganella, Janthinobacterium, Pseudomonas, Peribacillus, Psychrobacter* and *Sphingomonas*. The culturable bacterial genera *Cryobacterium, Janthinobacterium, Pseudomonas* and *Sphingomonas* of the present study have also been reported from Arctic cryoconites (Svalbard: Singh et al., 2014, Greenland: Perini et al., 2019; Singh et al., 2020). Genera such as *Pseudomonas, Cryobacterium* are of common occurrence in cryoconite habitats and have been previously reported from the Antarctic cryoconites (Christner et al., 2003). *Pseudomonas* has also been documented in the Alpine cryoconites (Lee et al., 2011). The occurrence of related phylotypes in geographically diverse cold environments suggests that they have unique adaptation strategies at low temperatures (Abyzov et al., 1998). These adaptation strategies include the occurrence of pigments, polyunsaturated and branched fatty acids (PUFAa) and or enzymes in the current isolates are active at low temperatures. Fatty acid composition of bacterial isolates of Hamtah glacier indicated presence of unsaturated and branched (including the iso- and anteiso-) fatty acids. Chintalapati et al. (2004) have also described the role of fatty acids in psychrophiles. Nishida and Murata (1996) reported the role of unsaturated and branched fatty acids in maintaining functional membrane fluidity at low temperatures. Thus, psychrophilic and psychrotolerant bacteria from glacier cryoconite adapt to the cold environment by preferentially synthesizing unsaturated and branched fatty acids. MiSeq high-throughput sequenced data of 16S rRNA genes showed presence of 440 genera of non-culturable bacteria in Hamtah glacier cryoconites (Supplementary Tables 4, 5). The major phyla present in current study are Proteobacteria, Actinobacteria, Bacteroidetes, Firmicutes, followed by Cyanobacteria, Chloroflexi, Acidobacteria, and Armatimonadetes were previously identified from Greenland cryoconites (Uetake et al., 2016). Additionally, the present study also showed seven common phyla such as Proteobacteria, Cyanobacteria, Bacteroidetes, Actinobacteria, Acidobacteria, Chloroflexi and Planctonycetes were also recorded from cryoconites of Svalbard, Arctic (Edwards et al., 2011). Himalayan cryoconites showed dominance of Alpha-, Betaproteobacteria is consistent with earlier studies from Antarctica (Christner et al., 2003). Deltaproteobacteria and Firmicutes were reported from majority of cryoconites holes resembles current observation (Cameron et al., 2012). The presence of eight genera of cyanobacteria (*Chroococcidiopsis, Chamaesiphon, Leptolyngbya, Microcoleus, Phormidesmis, Phormidium, Tychonema, Anabaenopsis, Nostoc, Calothrix*) and non-cyanobacterial photosynthetic bacteria *Rhodobacter* and *Chloroflexi* in current study may have an important role in primary production in Hamtah glacier cryoconites holes. Cyanobacterial taxa have also been identified globally in cryoconites of other cold places (Edwards et al., 2011; Uetake et al., 2016). The chemolithoautotrophic Nitrosomonadales genera *Nitrosomonas* and *Nitrosospira* has ability to oxidize ammonia to nitrite are present in current study were also reported from other cold habitat (Segawa et al., 2012). Besides this other *Nitrosomonadales taxa, Candidatus, Nitrotoga, Ferriphaselus, Gallionella, Sideroxydans* were observed in the present study (Supplementary Table 5). We also identified seven bacterial phyla from Hamtah glacier Snout (Actinobacteria, Bacteroidetes, Chloroflexi, Firmicutes, Proteobacteria, Spirochaetes and Verrucomicrobia), and six phyla from a glacier-stream/Nala (Acidobacteria, Actinobacteria, Bacteroidetes, Cyanobacteria, Firmicutes, Proteobacteria) which are consistent with an earlier report from Himalayan Kafni proglacial soils (Srinivas et al., 2011). Proteobacteria, Actinobacteria, Bacteroidetes and Firmicutes were well represented in all the sampling sites; however, some bacterial groups have shown uneven spatial distribution between sampling sites in the present study, resembling Arctic cryoconite holes (Edwards et al., 2011). Physico-chemical conditions of glaciers, topography, and aerial deposition of dust/propagules are possibly responsible for the spatial distribution pattern of bacteria in Polar cryoconite including the Himalayas. Additionally, Bacterial community composition also depends on bed rock type as reported from Antarctica (Tytgat et al., 2016).

The culturable bacterial community profiles were distinct from those found in the metagenomic community. The non-culturable approach studies document a more enormous assortment of bacteria (440 genera) than the culture-based approach (7 genera) in this study. However, there are some common genera in both approaches, but they are fewer in number. Similar observations have also been recorded from Arctic and Antarctic cryoconites (Christner et al., 2003; Edwards et al., 2011; Uetake et al., 2016). The culture based approach was applied in present study is to determine and compare physiological characteristics/biopotentials of individual species' of-glacier cryoconites. Furthermore, to understand the ecological roles (inter- and intra-species interactions, adaptation strategies etc.) culturable approach is very important (Jiang et al., 2006).

## Conclusions

Cryoconite holes are important microcosms on the glacier where cold-adapted microbes flourish. The present study focused on characterizing the bacterial diversity of Himalayan cryoconites holes through culture- and non-culture-based approaches. The culturable bacterial population in cryoconite holes is sizeable (seven genera), whereas the non-culturable population predominates with 440 genera. These are some of the dominant genera known to occur in the colder habitats of the world. Although the culturable bacterial communities in these microhabitats is limited, but strains differ widely in their antibiotic sensitivity characters. Carbon utilization tests showed that the bacterial isolates from cryoconite holes prefer simpler carbon sources. Bacterial isolates produce high amounts of polyunsaturated-branched fatty acids (PUFAs), and various cold active enzymes indicating the prospect of biotechnology in Himalaya. To detect the functions in whole bacterial communities of Himalayan cryoconites there is need of further studies on metatranscriptomics and DNA microarrays.

## Data availability statement

The datasets presented in this study can be found in online repositories. The names of the repository/repositories and accession number(s) can be found in the article/Supplementary material.

## Author contributions

PS: Writing – original draft, Methodology, Formal analysis. SS: Sampling, Data curation, Funding acquisition, Writing – review & editing. TS: Formal analysis (MiSeq data), Writing – review & editing. PKS: Writing – review & editing.

## Supplementary material

Supplementary Figure 1 **(a, b)** Landscape of Hamtah Glacier and **(c1–c4)** Cryoconite holes.
