# Supplementary figures and images for: Bacterial diversity and biopotentials of Hamtah glacier cryoconites, Himalaya
Source: Front Microbiol. 2024 May 1;15:1362678. doi: 10.3389/fmicb.2024.1362678 (PMC11094618; doi:10.3389/fmicb.2024.1362678)

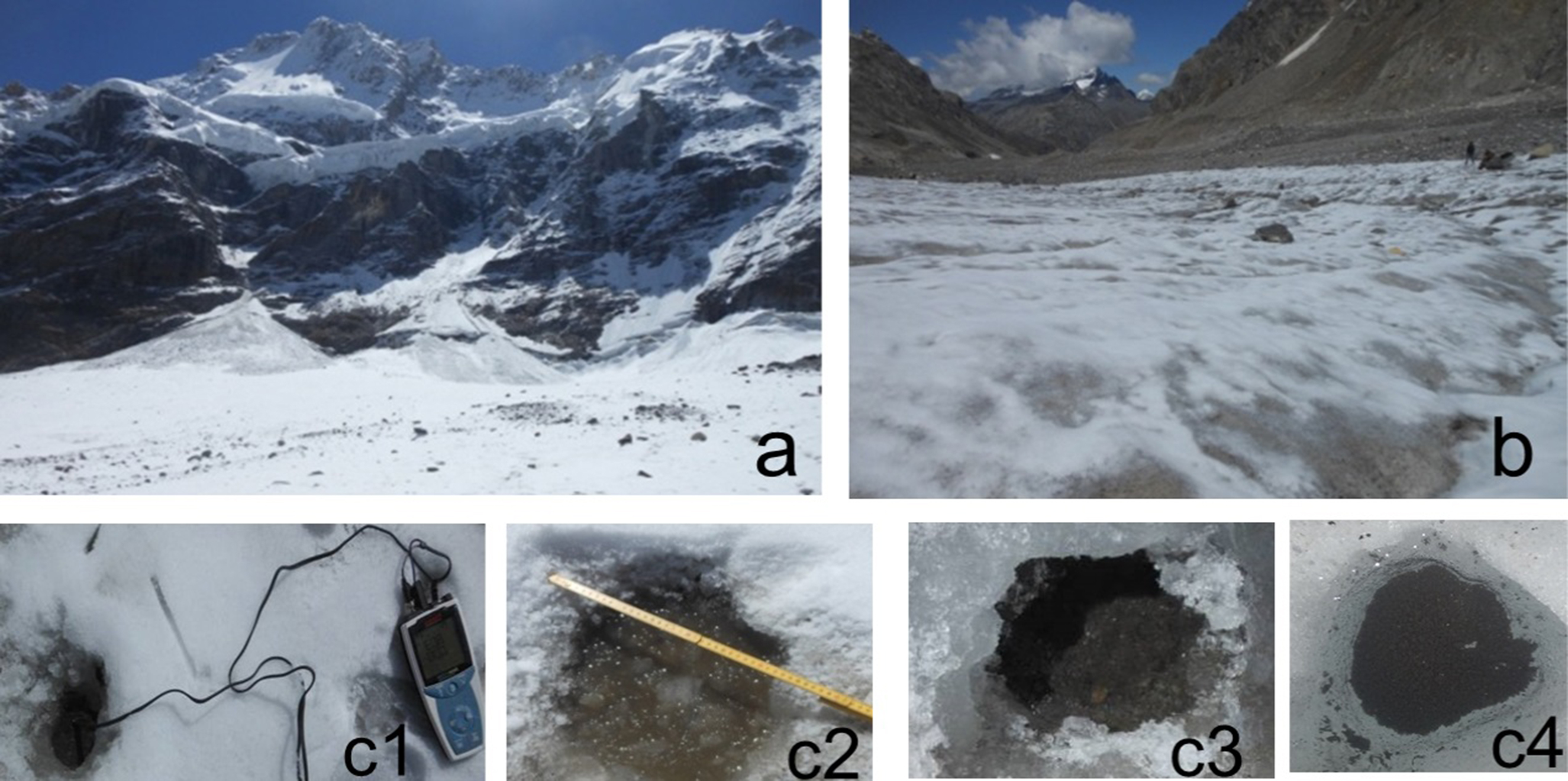

Supplement: Supplementary Figure 1 — (a, b) Landscape of Hamtah Glacier and (c1–c4) Cryoconite holes. [file Image_1.jpg]
