# Supplementary material for: Bacterial diversity and biopotentials of Hamtah glacier cryoconites, Himalaya
Source: Front Microbiol. 2024 May 1;15:1362678. doi: 10.3389/fmicb.2024.1362678 (PMC11094618; doi:10.3389/fmicb.2024.1362678)
Supplement: Supplementary file 3 [file Table_2.doc]

**Supplimentary Table 2.** Phenotypic characters (Carbon source utilization test) of the bacterial isolates

(**+** positive; W weak positive; **-** negative;)

| **Sr.No.** | **Carbohydrate** | **A2-6** *Cryobacterium arcticum* | **B2-3**  [*Janthinobacterium svalbardensis*](http://www.ezbiocloud.net/eztaxon/hierarchy?m=nomen_view&nid=Janthinobacterium+svalbardensis) | **B2-6** *[Pseudomonas simiae](http://blast.ncbi.nlm.nih.gov/Blast.cgi#alnHdr_255957414)* | **B2-8** [*Janthinobacterium svalbardensis*](http://www.ezbiocloud.net/eztaxon/hierarchy?m=nomen_view&nid=Janthinobacterium+svalbardensis) | **B2P3**  *[Pseudomonas simiae](http://blast.ncbi.nlm.nih.gov/Blast.cgi#alnHdr_255957414)* | **B2P4**  *[Pseudomonas simiae](http://blast.ncbi.nlm.nih.gov/Blast.cgi#alnHdr_255957414)* | **B2P6** *[Pseudomonas simiae](http://blast.ncbi.nlm.nih.gov/Blast.cgi#alnHdr_255957414)* | **B2p-7** *Sphingomonas glacialis* | **B2P11***[Pseudomonas simiae](http://blast.ncbi.nlm.nih.gov/Blast.cgi#alnHdr_255957414)* | **Ecry-2** [*Psychrobacter pulmonis*](http://www.ezbiocloud.net/eztaxon/hierarchy?m=nomen_view&nid=Psychrobacter+pulmonis) | **Ecry-4** *Peribacillus frigoritolerans* | **HF5**  [*Psychrobacter pulmonis*](http://www.ezbiocloud.net/eztaxon/hierarchy?m=nomen_view&nid=Psychrobacter+pulmonis) | **HF6**  *[Pseudomonas simiae](http://blast.ncbi.nlm.nih.gov/Blast.cgi#alnHdr_255957414)* |
| --- | --- | --- | --- | --- | --- | --- | --- | --- | --- | --- | --- | --- | --- | --- |
| 1. | Lactose | w | **-** | w | **⁻** | **-** | **-** | **-** | **⁻** | **-** | w | **+** | **-** | **-** |
| 2. | Xylose | **+** | **-** | **+** | **⁻** | **+** | **+** | **+** | w | **+** | w | **+** | **-** | **+** |
| 3. | Maltose | **+** | **-** | **⁻** | **⁻** | **-** | **-** | **-** | w | **-** | **⁻** | **⁻** | **-** | **-** |
| 4. | Fructose | **+** | **-** | w | **⁻** | **-** | **-** | **-** | **+** | **-** | **⁻** | **⁻** | **-** | **-** |
| 5. | Dextrose | **+** | **-** | **+** | **⁻** | **+** | **+** | **+** | **+** | **+** | **+** | **+** | **-** | **+** |
| 6. | Galactose | **+** | **-** | **+** | **⁻** | **+** | **+** | **+** | w | **+** | w | w | **-** | **+** |
| 7. | Raffinose | **+** | **-** | w | **⁻** | **-** | **-** | **-** | **⁻** | **-** | w | w | **-** | **-** |
| 8. | Trehalose | **+** | **-** | w | **⁻** | **-** | **-** | **-** | **⁻** | **-** | w | w | **-** | **-** |
| 9. | Melibiose | **+** | **-** | **+** | **⁻** | **+** | **+** | **+** | **⁻** | **+** | w | w | **-** | **+** |
| 10. | Sucrose | **+** | **-** | w | **⁻** | **+** | **+** | **+** | w | **+** | **⁻** | **⁻** | **-** | **+** |
| 11. | L-Arabinose | **+** | **-** | **+** | **⁻** | **+** | **+** | **-** | **+** | **+** | w | **+** | **-** | **+** |
| 12. | Mannose | **+** | **-** | **+** | **⁻** | **+** | **+** | **+** | **+** | **+** | **+** | **+** | **-** | **+** |
| 13. | Insulin | **+** | **-** | w | **⁻** | **-** | **-** | **-** | w | **-** | w | **⁻** | **-** | **-** |
| 14. | Sodium gluconate | w | **-** | w | **⁻** | **-** | **-** | **-** | w | **-** | w | w | **-** | **-** |
| 15. | Glycerol | w | **-** | w | **⁻** | **+** | **-** | **-** | w | **+** | w | w | **-** | **+** |
| 16. | Salicin | **+** | **-** | w | **⁻** | **-** | **-** | **+** | **⁻** | **-** | w | w | **-** | **-** |
| 17. | Dulcitol | **⁻** | **-** | w | **⁻** | **-** | **-** | **-** | **⁻** | **-** | **⁻** | w | **-** | **-** |
| 18. | Inositol | **⁻** | **-** | w | **⁻** | **-** | **-** | **-** | **⁻** | **-** | w | w | **-** | **-** |
| 19. | Sorbitol | **⁻** | **-** | w | **⁻** | **-** | **-** | **-** | **⁻** | **-** | w | **⁻** | **-** | **+** |
| 20. | Mannitol | **+** | **-** | w | **⁻** | **-** | **-** | **-** | **+** | **-** | **⁻** | **⁻** | **-** | **+** |
| 21. | Adonitol | **⁻** | **-** | w | **⁻** | **-** | **-** | **-** | **⁻** | **-** | w | w | **-** | **-** |
| 22. | Arabitol | **⁻** | **-** | w | **⁻** | **-** | **-** | **-** | w | **-** | **⁻** | **⁻** | **-** | **-** |
| 23. | Erythritol | **⁻** | **-** | **⁻** | **⁻** | **-** | **-** | **-** | **⁻** | **-** | w | w | **-** | **-** |
| 24. | α-methyl-D-glucoside | **⁻** | **-** | w | **⁻** | **-** | **-** | **-** | **⁻** | **-** | w | w | **-** | **-** |
| 25. | Rhamnose | w | **-** | w | **⁻** | **-** | **-** | **-** | **⁻** | **-** | w | w | **-** | **-** |
| 26. | Cellobiose | **+** | **-** | **⁻** | **⁻** | **-** | **-** | **-** | w | **-** | **+** | **+** | **-** | **-** |
| 27. | Melezitose | **+** | **-** | **⁻** | **⁻** | **-** | **-** | **-** | **⁻** | **-** | w | w | **-** | **-** |
| 28. | α-methyl-D-mannoside | **⁻** | **-** | **⁻** | **⁻** | **-** | **-** | **-** | **⁻** | **-** | w | w | **-** | **-** |
| 29. | Xylitol | w | **-** | **+** | **⁻** | **-** | **-** | **-** | **⁻** | **-** | w | w | **-** | **-** |
| 30. | ONPG | **+** | **+** | **⁻** | **+** | **-** | **-** | **-** | **+** | **-** | **⁻** | **⁻** | **-** | **-** |
| 31. | Esculin hydrolysis | **+** | **+** | **+** | **+** | **+** | **-** | **+** | **+** | **-** | **⁻** | **⁻** | **+** | **-** |
| 32. | D-Arabinose | **+** | **-** | **+** | **⁻** | **-** | **+** | **-** | **+** | **-** | **+** | **+** | **-** | **+** |
| 33. | Citrate utilization | **⁻** | **-** | **⁻** | **+** | **+** | **+** | **+** | **⁻** | **+** | **+** | **+** | **+** | **+** |
| 34. | Malonate utilization | **⁻** | **+** | **⁻** | **+** | **+** | **+** | **+** | **⁻** | **+** | **+** | **+** | **+** | **+** |
| 35. | Sorbose | **⁻** | **-** | **⁻** | **⁻** | **-** | **-** | **-** | **⁻** | **-** | w | **⁻** | **-** | **-** |
