# Supplementary material for: Bacterial diversity and biopotentials of Hamtah glacier cryoconites, Himalaya
Source: Front Microbiol. 2024 May 1;15:1362678. doi: 10.3389/fmicb.2024.1362678 (PMC11094618; doi:10.3389/fmicb.2024.1362678)
Supplement: Supplementary file 4 [file Table_3.doc]

**Supplementary Table 3** Extracellular enzymatic activity of different bacterial strains of Hamtah glacier cryoconite, Himalaya.

| **Sr. No.** | **Culture**  **code** | **Protease** | | | **Amylase** | | | **Lipase** | | | **Cellulase** | | |
| --- | --- | --- | --- | --- | --- | --- | --- | --- | --- | --- | --- | --- | --- |
|  |  | **4^o^C** | **15^o^C** | **22^o^C** | **4^o^C** | **15^o^C** | **22^o^C** | **4^o^C** | **15^o^C** | **22^o^C** | **4^o^C** | **15^o^C** | **22^o^C** |
| 1 | A2-6 | - | - | - | - | - | - | ++ | + | ++ | ++ | ++ | ++ |
| 2 | A2(2) | - | - | - | - | - | - | - | - | - | - | - | - |
| 3 | A2-7 | - | - | - | - | - | - | - | - | - | ++ | - | - |
| 4 | A4-5 | - | - | - | - | - | - | - | - | - | - | - | - |
| 5 | A4P-1 | w | - | - | - | - | - | - | - | - | - | - | - |
| 6 | A4P-4 | - | - | - | - | - | - | w | - | - | - | - | - |
| 7 | A4P-5 | - | - | - | - | - | - | - | - | - | - | - | - |
| 8 | A4P-6 | - | - | - | - | - | - | - | - | - | - | - | - |
| 9 | A4P-7 | - | - | - | - | - | - | w | - | - | - | - | - |
| 10 | B2(f) | - | - | - | - | - | - | - | - | - | - | - | - |
| 11 | B2(G) | - | - | - | - | - | - | - | - | - | - | - | - |
| 12 | B2-3 | w | - | - | - | - | - | - | - | - | - | - | - |
| 13 | B2-6 | ++ | + | w | - | - | - | - | - | - | - | ++ | - |
| 14 | B2-8 | w | w | - | - | - | - | W | w | w | w | - | - |
| 15 | B2-12 | + | w | - | - | - | - | - | - | - | - | - | - |
| 16 | B2-14 | w | w | - | - | - | - | - | - | - | - | - | - |
| 17 | B2P1 | - | w | - | - | - | - | - | - | - | - | - | - |
| 18 | B2P3 | ++ | ++ | + | - | - | - | ++ | - | - | ++ | + | ++ |
| 19 | B2P4 | ++ | ++ | + | - | - | - | + | - | - | ++ | + | ++ |
| 20 | B2P6 | + | ++ | + | - | - | - | + | - | - | ++ | + | ++ |
| 21 | B2-P7 | - | - | - | - | - | - | - | w | - | - | - | - |
| 22 | B2P9 | w | - | - | - | - | - | w | w | - | - | - | - |
| 23 | B2P10 | - | - | - | - | - | - | - | - | - | - | - | - |
| 24 | B2P11 | ++ | + | + | w | w | w | + | - | - | ++ | ++ | ++ |
| 25 | B2P13 | - | - | - | - | - | - | - | - | - | - | - | - |
| 26 | B2P14 | - | - | - | - | - | - | - | - | - | - | - | - |
| 27 | B2P15 | - | - | - | - | - | - | - | - | - | - | - | - |
| 28 | EI-1 | - | - | - | - | - | - | W | - | - | - | - | - |
| 28 | EI-2 | - | - | - | - | - | - | W | - | - | - | - | - |
| 29 | EI-3 | - | - | - | - | - | - | W | - | - | - | - | - |
| 30 | EI-4 | - | - | - | - | - | - | - | - | - | - | - | - |
| 31 | EI-5 | - | - | - | - | - | - | - | - | - | - | - | - |
| 32 | EI-6 | - | - | - | - | - | - | - | - | - | - | - | - |
| 33 | EI-8 | w | - | - | - | - | - | - | - | - | - | - | - |
| 34 | EI-9 | + | - | - | - | - | - | - | - | - | - | - | - |
| 35 | EI-10 | + | - | - | - | - | - | - | - | - | - | - | - |
| 36 | EI-11 | w | - | - | - | - | - | - | - | - | - | - | - |
| 37 | EI-13 | - | - | - | - | - | - | - | - | - | - | - | - |
| 38 | EI-16 | - | - | - | - | - | - | - | - | - | - | - | - |
| 39 | EI-17 | - | - | - | - | - | - | - | - | - | - | - | - |
| 39 | E-cry2 | - | - | - | - | - | - | - | - | - | - | - | - |
| 40 | E-cry4 | - | - | w | - | - | + | - | w | + | + | W | + |
| 41 | E-cry7 | w | - | - | - | - | - | - | - | - | - | - | - |
| 42 | HF5 | - | - | - | - | - | - | - | - | - | - | - | - |
| 43 | HF6 | ++ | + | + | - | - | + | - | - | - | + | + | + |
| 44 | HF8 | ++ | ++ | ++ | - | - | + | - | - | - | ++ | + | + |
| 45 | HF9 | ++ | + | + | - | - | - | - | - | - | + | ++ | ++ |
