# Supplementary material for: Bacterial diversity and biopotentials of Hamtah glacier cryoconites, Himalaya
Source: Front Microbiol. 2024 May 1;15:1362678. doi: 10.3389/fmicb.2024.1362678 (PMC11094618; doi:10.3389/fmicb.2024.1362678)
Supplement: Supplementary file 5 [file Table_4.docx]

**Supplementary Table 4** Composition of Non-culturable bacteria isolated from Hamtah glacier cryoconites, snout and stream (nala), Himalaya.

| **Phyla/Sub-Phyla** | **Order** | **Family** | **Genera** |
| --- | --- | --- | --- |
| **Acidobacteria** | Acidobacteriales | Acidobacteriaceae | *Acidobacterium, Granulicella, Terriglobus* |
|  | Holophagales (Holophagae) | Holophagaceae | *Geothrix, Holophaga* |
| **Actinobacteria** | Acidimicrobiales (Acidimicrobiia) | Iamiaceae | *Iamia* |
|  | Corynebacteriales | Corynebacteriaceae | *Corynebacterium* |
|  |  | Mycobacteriaceae | *Mycobacterium* |
|  |  | Nocardiaceae | *Rhodococcus* |
|  | Frankiales | Cryptosporangiaceae | *Fodinicola* |
|  |  | Frankiaceae | *Frankia, Jatrophihabitans* |
|  |  | Geodermatophilaceae | *Blastococcus, Geodermatophilus* |
|  |  | Nakamurellaceae | *Nakamurella* |
|  |  | Sporichthyaceae | *Sporichthya* |
|  | Kineosporiales | Kineosporiaceae | *Angustibacter, Kineosporia* |
|  | Micrococcales | Cellulomonadaceae | *Actinotalea, Cellulomonas* |
|  |  | Demequinaceae | *Demequina, Lysinimicrobium* |
|  |  | Dermabacteraceae | *Brachybacterium, Kytococcus* |
|  |  | Intrasporangiaceae | *Fodinibacter, Humibacillus, Intrasporangium, Janibacter, Knoellia, Lapillicoccus, Oryzihumus, Terrabacter,* |
|  |  | Microbacteriaceae | *Agromyces, Alpinimonas, Amnibacterium, Clavibacter, Cryobacterium, Curtobacterium, Diaminobutyricimonas, Frigoribacterium, Frondihabitans, Glaciibacter, Leifsonia, Leucobacter, Lysinimonas, Marisediminicola, Microbacterium, Mycetocola, Plantibacter, Pseudoclavibacter, Rhodoglobus, Salinibacterium, Schumannella, Subtercola, Yonghaparkia* |
|  |  | Micrococcaceae | *Arthrobacter, Micrococcus, Rothia,* |
|  |  | Promicromonosporaceae | *Cellulosimicrobium, Xylanimicrobium* |
|  | Micromonosporales | Micromonosporaceae | *Actinoplanes, Luedemannella, Micromonospora, Pilimelia, Plantactinospora, Polymorphospora, Verrucosispora,* |
|  | Propionibacteriales | Nocardioidaceae | *Aeromicrobium, Marmoricola, Nocardioides* |
|  |  | Propionibacteriaceae | *Aestuariimicrobium, Friedmanniella, Microlunatus, Propionibacterium* |
|  | Pseudonocardiales | Pseudonocardiaceae | *Actinomycetospora, Crossiella, Pseudonocardia,* |
|  | Streptomycetales | Streptomycetaceae | *Streptomyces* |
|  | Streptosporangiales | Streptosporangiaceae | *Nonomuraea, Sinosporangium* |
|  | Coriobacteriales (Coriobacteriia) | Coriobacteriaceae | *Collinsella* |
|  | Rubrobacterales (Rubrobacteria) | Rubrobacteriaceae | *Rubrobacter* |
|  | Gaiellales (Thermoleophilia) | Gaiellaceae | *Gaiella* |
|  | Solirubrobacterales (Thermoleophilia) | Patulibacteraceae | *Patulibacter* |
|  |  | Solirubrobacteraceae | *Solirubrobacter* |
| **Armatimonadetes** | Armatimonadales (Armatimonadia) | Armatimonadaceae | *Armatimonas* |
|  | Chthonomonadales (Chthonomonadetes) | Chthonomonadaceae | *Chthonomonas* |
| **Bacteroidetes** | Bacteroidales (Bacteroidia) | Bacteroidaceae | *Bacteroides* |
|  |  | Marinilabiaceae | *Mangroviflexus, Marinifilum,* |
|  |  | Porphyromonadaceae | *Dysgonomonas, Macellibacteroides, Odoribacter, Paludibacter, Parabacteroides, Petrimonas, Proteiniphilum,* |
|  |  | Prevotellaceae | *Alloprevotella, Prevotella,* |
|  |  | Rikenellaceae | *Alistipes* |
|  | Cytophagales (Cytophagia) | Cytophagaceae | *Arcicella, Chryseolinea, Cytophaga, Dyadobacter, Emticicia, Fibrella, Hymenobacter, Rhodocytophaga, Siphonobacter, Spirosoma, Sporocytophaga* |
|  |  | Flammeovirgaceae | *Cesiribacter, Flexithrix* |
|  | Flavobacteriales (Flavobacteriia) | Cryomorphaceae | *Fluviicola, Owenweeksia,* |
|  |  | Flavobacteriaceae | *Chryseobacterium, Cloacibacterium, Elizabethkingia, Flavobacterium, Myroides, Polaribacter,* |
|  | Sphingobacteriales (Sphingobacteriia) | Chitinophagaceae | *Chitinophaga, Ferruginibacter, Flavihumibacter, Flavisolibacter, Flavitalea, Hydrotalea, Lacibacter, Sediminibacterium, Segetibacter, Taibaiella,* |
|  | Sphingobacteriales (Sphingobacteriia) | Saprospiraceae | *Lewinella* |
|  |  | Sphingobacteriaceae | *Arcticibacter, Mucilaginibacter, Nubsella, Pedobacter, Solitalea, Sphingobacterium,* |
| **Caldiserica** | Caldisericales (Caldisericia) | Caldisericaceae | *Caldisericum* |
| **Chlamydiae** | Chlamydiales | Parachlamydiaceae | *Neochlamydia, Parachlamydia* |
|  |  | Waddliaceae | *Waddlia* |
| **Chloroflexi** | Anaerolineales (Anaerolineae) | Anaerolineaceae | *Anaerolinea, Leptolinea,* |
|  | Caldilineales (Caldilineae) | Caldilineaceae | *Caldilinea* |
|  | Chloroflexales (Chloroflexia) | Chloroflexaceae | *Chloronema* |
|  |  | Roseiflexaceae | *Roseiflexus* |
|  | Herpetosiphonales | Herpetosiphonaceae | *Herpetosiphon* |
|  | Ktedonobacterales (Ktedonobacteria) | Ktedonobacteraceae | *Ktedonobacter* |
|  |  | Thermosporotrichaceae | *Thermosporothrix* |
| **Cyanobacteria** | Sub sectionII | FamilyII | *Chroococcidiopsis* |
|  | Subsection III | FamilyI | *Chamaesiphon* |
|  | Subsection III | FamilyI | *Leptolyngbya* |
|  | Subsection III | FamilyI | *Microcoleus* |
|  | Subsection III | FamilyI | *Phormidesmis* |
|  | Subsection III | FamilyI | *Phormidium* |
|  | Subsection III | FamilyI | *Tychonema* |
|  | Subsection IV | Family I | *Anabaenopsis* |
|  | Subsection IV | Family I | *Nostoc* |
|  | Subsection IV | Family II | *Calothrix* |
| **Deferribacteres** | Deferribacterales (Deferribacteres) | Deferribacteraceae | *Denitrovibrio* |
| **Deinococcus-Thermus** | Deinococcales (Deinococci) | Deinococcaceae | *Deinococcus* |
|  |  | Trueperaceae | *Truepera* |
|  | Thermales | Thermaceae | *Thermus* |
| **Elusimicrobia** | Elusimicrobiales | Elusimicrobiaceae_ | *Elusimicrobium* |
| **Firmicutes** | Bacillales (Bacilli) | Alicyclobacillaceae | *Tumebacillus* |
|  |  | Bacillaceae | *Bacillus, Gracilibacillus, Lentibacillus, Oceanobacillus, Ornithinibacillus, Paucisalibacillus, Terribacillus,* |
|  |  | Paenibacillaceae | *Brevibacillus, Cohnella, Fontibacillus, Oxalophagus, Paenibacillus,* |
|  |  | Planococcaceae | *Bhargavaea, Chungangia, Lysinibacillus,*  *Solibacillus, Sporosarcina* |
|  |  | Sporolactobacillaceae | *Sporolactobacillus* |
|  |  | Staphylococcaceae | *Macrococcus* |
|  |  | Thermoactinomycetaceae | *Melghirimyces, Planifilum, Shimazuella, Thermoactinomyces,* |
|  | Lactobacillales | Streptococcaceae | *Streptococcus* |
|  | Clostridiales (Clostridia) | Christensenellaceae | *Christensenella* |
|  |  | Clostridiaceae | *Caloramator, Clostridium, Fonticella, Oxobacter, Proteiniclasticum,* |
|  |  | Eubacteriaceae | *Acetobacterium* |
|  |  | Gracilibacteraceae | *Lutispora* |
|  |  | Lachnospiraceae | *Blautia, Pseudobutyrivibrio* |
|  |  | Peptococcaceae | *Cryptanaerobacter, Desulfitibacter, Desulfosporosinus, Pelotomaculum, Thermincola* |
|  |  | Ruminococcaceae | *Faecalibacterium, Fastidiosipila, Saccharofermentans, Subdoligranulum* |
|  |  | Syntrophomonadaceae | *Syntrophomonas* |
|  | Thermoanaerobacterales | Thermoanaerobacteraceae | *Gelria* |
|  | Erysipelotrichales (Erysipelotrichia) | Erysipelotrichaceae | *Erysipelothrix, Turicibacter* |
|  | Selenomonadales (Negativicutes) | Veillonellaceae | *Anaerospora, Megamonas, Pelosinus, Sporomusa* |
| **Fusobacteria** | Fusobacteriales (Fusobacteriia) | Fusobacteriaceae | *Fusobacterium* |
| **Gemmatimonadetes** | Gemmatimonadales | Gemmatimonadaceae | *Gemmatimonas* |
| **Lentisphaerae** | Victivallales (Lentisphaeria) | Victivallaceae | *Victivallis* |
| **Nitrospirae** | Nitrospirales (Nitrospira) | Nitrospiraceae | *Nitrospira* |
| **Planctomycetes** | Planctomycetales (Planctomycetacia) | Planctomycetaceae | *Gemmata, Isosphaera, Pirellula, Planctomyces, Schlesneria, Singulisphaera* |
| **Proteobacteria**  Alphaproteobacteria | Caulobacterales | Caulobacteraceae | *Asticcacaulis, Brevundimonas, Caulobacter, Phenylobacterium* |
|  |  | Hyphomonadaceae | *Hirschia, Woodsholea* |
|  | Magnetococcales | Magnetococcaceae | *Magnetococcus* |
|  | Rhizobiales | Aurantimonadaceae | *Fulvimarina,* |
|  |  | Beijerinckiaceae | *Beijerinckia, Methylorosula, Methylovirgula* |
|  |  | Bradyrhizobiaceae | *Afipia, Bosea, Bradyrhizobium, Rhodopseudomonas, Salinarimonas, Tardiphaga,* |
|  |  | Brucellaceae | *Ochrobactrum* |
|  |  | Hyphomicrobiaceae | *Devosia, Pedomicrobium* |
|  |  | Rhodobacter | *Rhodobacter* |
|  |  | Methylocystaceae | *Pleomorphomonas* |
|  |  | Skermanella | *Skermanella* |
|  |  | Rhizobiaceae | *Rhizobium* |
|  |  | Rhodobiaceae | *Rhodobium* |
|  |  | Xanthobacteraceae | *Pseudolabrys* |
|  | Rhodobacterales | Rhodobacteraceae | *Amaricoccus, Gemmobacter, Rhodobacter, Roseovarius, Rubellimicrobium, Tabrizicola* |
|  | Rhodospirillales | Acetobacteraceae | *Acidicaldus, Acidiphilium, Acidisphaera, Acidocella, Belnapia, Rhodovarius, Roseococcus, Roseomonas, Rubritepida* |
|  |  | Rhodospirillaceae | *Dongia, Inquilinus, Rhodovibrio, Skermanella, Thalassospira,* |
|  | Rickettsiales | Rickettsiaceae | *Rickettsia* |
|  | Sphingomonadales | Sphingomonadaceae | *Novosphingobium, Sandarakinorhabdus, Sphingobium, Sphingomonas, Sphingopyxis, Sphingorhabdus, Zymomonas* |
| Betaproteobacteria | Burkholderiales | Alcaligenaceae | *Alcaligenes, Derxia, Sutterella,* |
|  |  | Burkholderiaceae | *Burkholderia, Limnobacter, Pandoraea, Ralstonia,* |
|  |  | Comamonadaceae | *Acidovorax, Albidiferax, Aquabacterium, Aquincola, Caenimonas, Caldimonas, Chlorochromatium, Comamonas, Curvibacter, Delftia, Hydrogenophaga, Hylemonella, Leptothrix, Limnohabitans, Malikia, Methylibium, Mitsuaria, Paucibacter, Pelomonas, Piscinibacter, Polaromonas, Ramlibacter, Rhizobacter, Rhodoferax, Roseateles, Rubrivivax, Sphaerotilus, Variovorax, Xenophilus, Xylophilus* |
|  |  | Oxalobacteraceae | *Actimicrobium, Collimonas,* ***Duganella****, Glaciimonas, Herbaspirillum, Herminiimonas,* ***Janthinobacterium****, Massilia, Noviherbaspirillum, Oxalicibacterium, Paucimonas, Rugamonas, Telluria, Undibacterium,* |
|  | Hydrogenophilales | Hydrogenophilaceae | *Ferritrophicum, Sulfuricella, Thiobacillus,* |
|  | Methylophilales | Methylophilaceae | *Methylobacillus, Methylophilus, Methylotenera,* |
|  | Neisseriales | Neisseriaceae | *Jeongeupia, Laribacter, Leeia, Neisseria,* |
|  | Nitrosomonadales | Gallionellaceae | *Ferriphaselus, Gallionella, Sideroxydans,* |
|  |  | Nitrosomonadaceae | *Nitrosomonas, Nitrosospira,* |
|  | Procabacteriales | Procabacteriaceae | *Procabacter* |
|  | Rhodocyclales | Rhodocyclaceae | *Azoarcus, Dechloromonas, Denitratisoma, Ferribacterium, Georgfuchsia, Propionivibrio, Sterolibacterium, Sulfuritalea, Uliginosibacterium, Zoogloea,* |
| Deltaproteobacteria | Bdellovibrionales | Bacteriovoracaceae | *Bacteriovorax, Peredibacter* |
|  |  | Bdellovibrionaceae | *Bdellovibrio* |
|  | Desulfarculales | Desulfarculaceae | *Desulfarculus,* |
|  |  | Desulfobacteraceae | *Desulfonema,* |
|  | Desulfobacterales | Desulfobulbaceae | *Desulfobulbus, Desulfocapsa, Desulfurivibrio,* |
|  |  | Nitrospinaceae | *Nitrospina* |
|  | Desulfovibrionales | Desulfohalobiaceae | *Desulfothermus, Desulfovermiculus* |
|  |  | Desulfovibrionaceae | *Desulfovibrio* |
|  | Desulfuromonadales | Desulfuromonadaceae | *Desulfuromonas, Pelobacter* |
|  |  | Geobacteraceae | *Geobacter* |
|  | Myxococcales | Cystobacteraceae | *Anaeromyxobacter* |
|  |  | Haliangiaceae | *Haliangium* |
|  |  | Phaselicystidaceae | *Phaselicystis* |
|  |  | Polyangiaceae | *Byssovorax, Sorangium* |
|  |  | Sandaracinaceae | *Sandaracinus* |
|  | Syntrophobacterales | Syntrophaceae | *Desulfobacca, Smithella* |
| Epsilonproteobacteria | Campylobacterales | Campylobacteraceae | *Arcobacter, Sulfurospirillum, Thiofractor,* |
|  |  | Helicobacteraceae | *Sulfuricurvum, Sulfurimonas* |
|  | Nautiliales | Nautiliaceae | *Nitratifractor* |
| Gammaproteobacteria | Acidithiobacillales | Acidithiobacillaceae | *Acidithiobacillus* |
|  | Aeromonadales | Aeromonadaceae | *Tolumonas* |
|  | Alteromonadales | Pseudoalteromonadaceae | *Pseudoalteromonas* |
|  | Chromatiales | Ectothiorhodospiraceae | *Acidiferrobacter, Halorhodospira, Thioalkalispira* |
|  | Enterobacteriales | Enterobacteriaceae | *Arsenophonus, Brenneria, Citrobacter, Enterobacter, Erwinia, Ewingella, Klebsiella, Pantoea, Pectobacterium, Rahnella, Raoultella, Salmonella, Tatumella,* |
|  | Legionellales | Coxiellaceae | *Aquicella, Coxiella,* |
|  |  | Legionellaceae | *Legionella* |
|  | Oceanospirillales | Alcanivorax | *Alcanivorax* |
|  |  | Oceanospirillaceae | *Marinobacterium* |
|  | Pasteurellales | Pasteurellaceae | *Aggregatibacter* |
|  | Pseudomonadales | Moraxellaceae | *Acinetobacter, Alkanindiges, Enhydrobacter, Perlucidibaca,* |
|  |  | Pseudomonadaceae | *Cellvibrio,* ***Pseudomonas*** |
|  | Thiotrichales | Piscirickettsiaceae | *Methylophaga, Sulfurivirga* |
|  | Thiotrichales | Thiotrichaceae | *Methylohalomonas* |
|  | Xanthomonadales | Xanthomonadaceae | *Arenimonas, Dokdonella, Dyella, Luteimonas, Lysobacter, Pseudofulvimonas, Pseudoxanthomonas, Rhodanobacter, Silanimonas, Stenotrophomonas, Thermomonas, Xanthomonas, Xylella,* |
| **Spirochaetae** | Spirochaetales (Spirochaetes) | Leptospiraceae | *Turneriella* |
|  |  | Spirochaetaceae | *Spirochaeta, Treponema* |
| **Verrucomicrobia** | Opitutales (Opitutae) | Opitutaceae | *Opitutus* |
|  | Puniceicoccales | Puniceicoccaceae | *Cerasicoccus* |
|  | Chthoniobacterales (Spartobacteria) | Chthoniobacteraceae | *Chthoniobacter* |
|  | Verrucomicrobiales (Verrucomicrobiae) | Verrucomicrobiaceae | *Prosthecobacter, Roseibacillus* |
