# Supplementary material for: Bacterial diversity and biopotentials of Hamtah glacier cryoconites, Himalaya
Source: Front Microbiol. 2024 May 1;15:1362678. doi: 10.3389/fmicb.2024.1362678 (PMC11094618; doi:10.3389/fmicb.2024.1362678)
Supplement: Supplementary file 7 [file Table_6.doc]

**Supplementary Table 6**. Cellular fatty acid composition of different species of Hamtah glacier cryoconites, Himalaya

.

| **Fatty acid composition** | **Gram-positive bacteria** | | | | | | **Gram-negative bacteria** | | | | | |
| --- | --- | --- | --- | --- | --- | --- | --- | --- | --- | --- | --- | --- |
| *Peribacillus frigoritolerans* **Ecry4** (MF467864) | | *Cryobacterium arcticum* **A_2_6** (KY783365) | | *Sphingomonas glacialis* **B_2_P-7** (MF467873) | | [*Psychrobacter pulmonis*](http://www.ezbiocloud.net/eztaxon/hierarchy?m=nomen_view&nid=Psychrobacter+pulmonis) **Ecry2** (KY783397) | | *[Pseudomonas](http://blast.ncbi.nlm.nih.gov/Blast.cgi#alnHdr_255957414) marginalis* **B_2_6 (**KY783373) | | [*Janthinobacterium svalbardensis*](http://www.ezbiocloud.net/eztaxon/hierarchy?m=nomen_view&nid=Janthinobacterium+svalbardensis)**B28** (KY783374) | |
| **Growth Medium** | **NA** (Mean) | **TSBA** (Mean) | **NA** (Mean) | **TSBA** (Mean) | ABM (Mean) | **TSBA** (Mean) | **NA** (Mean) | **TSBA** (Mean) | **ZMA** (Mean) | **TSBA** (Mean) | **NA** (Mean) | **TSBA** (Mean) |
| **Saturated Fatty acids** |  |  |  |  |  |  |  |  |  |  |  |  |
| C_9:0_ | 0.48 |  |  |  |  |  | 0.5 | 0.44 |  |  |  |  |
| C_10:0_ | 3.59 | 3.94 |  |  |  |  | 3.88 | 4.82 | 0.15 | 0.27 | 0.37 | 0.33 |
| C_10:0_ 3OH |  |  |  |  |  |  |  |  | 3.86 | 3.82 | 4.46 | 4.06 |
| C_12:0_ | 1.80 | 2.00 |  |  |  |  | 1.82 | 2.26 | 2.84 | 3.10 | 4.21 | 3.86 |
| C _12:0_ 2OH |  |  |  |  |  |  |  |  | 3.86 | 3.82 | 2.13 | 1.53 |
| C _12:0_ 3OH | 12.35 | 14.2 |  |  |  |  | 4.85 | 5.81 | 4.67 | 4.96 |  |  |
| C_14:0_ |  |  | 0.14 | 0.17 | 0.21 |  |  |  | 0.64 | 0.31 | 0.48 | 0.72 |
| C_16:0_ | 1.69 | 3.69 | 2.55 | 2.94 | 2.41 | 2.15 | 1.6 | 2.55 | 18.96 | 20.32 | 29.59 | 25.95 |
| C_17:0_ | 0.36 |  | 0.88 |  |  |  |  | 0.30 |  |  |  |  |
| C_18:0_ | 5.44 | 3.53 | 0.31 | 0.47 |  | 0.24 | 1.83 | 2.66 | 0.48 | 0.44 |  | 0.41 |
| ***Total*** | **25.71** | **27.36** | **3.88** | **3.58** | **2.62** | **2.39** | **14.48** | **18.84** | **35.46** | **37.04** | **41.24** | **36.45** |
| **Branched** |  |  |  |  |  |  |  |  |  |  |  |  |
| **Iso-branched chain** |  |  |  |  |  |  |  |  |  |  |  |  |
| iso-C_14:0_ |  |  | 0.35 | 0.71 | 1.59 | 0.85 |  |  |  |  |  |  |
| iso-C_15:0_ |  |  | 0.36 | 0.39 | 2.92 | 1.12 |  |  |  |  |  |  |
| Iso-C_15:1_ G |  |  | 5.50 | 7.16 | 2.74 | 3.59 |  |  |  |  |  |  |
| iso-C_16:0_ |  |  | 18.15 | 21.21 | 21.87 | 15.03 |  |  |  |  |  |  |
| Iso-C_16:1_ G |  |  | 0.29 | 0.46 |  |  |  |  |  |  |  |  |
| iso-C_17:0_ | 0.59 | 0.55 | 0.23 | 0.29 | 0.41 | 0.30 | 0.66 | 0.58 |  |  |  |  |
| **Anteiso-branched chain** |  |  |  |  |  |  |  |  |  |  |  |  |
| anteiso-C_15:0_ |  |  | 46.23 | 44.53 | 54.02 | 56.44 |  |  |  |  |  |  |
| Anteiso-C_15:1_  AG |  |  | 2.87 | 2.22 |  |  |  |  |  |  |  |  |
| anteiso-C_17:0_ |  |  | 22.49 | 19.62 | 10.52 | 15.85 |  |  |  |  |  |  |
| Summed Feature 1  15:1 iso H/13:0 3OH |  |  |  |  |  |  | 0.67 | 0.7 |  |  |  |  |
| Sum In Feature 2: 16:1 iso I/14:0 3OH | 1.56 | 1.76 |  |  |  |  | 1.52 | 1.79 |  |  |  |  |
| ^#^Sum In Feature 5 18:0 ante/18:2 w6,9c |  |  |  |  |  |  |  |  | 0.23 |  | 0.54 | 0.17 |
| ***Total*** | **2.15** | **2.31** | **96.47** | **96.59** | **94.07** | **93.18** | **2.85** | **3.07** | **0.23** |  | **0.54** | **0.17** |
| **Unsaturated Fatty acids** |  |  |  |  |  |  |  |  |  |  |  |  |
| C_12:1_ 3OH |  |  |  |  |  |  |  |  |  | 0.18 |  |  |
| C_18:1_ 2OH |  |  |  |  |  |  |  |  |  |  | 0.16 |  |
| 16:1 w5c |  |  |  |  |  |  |  |  | 0.12 |  | 0.25 | 0.36 |
| 17:1 w7c |  |  |  |  |  |  |  |  |  |  | 0.21 |  |
| 17:1 w8c | 6.38 |  |  |  |  |  | 4.82 |  |  |  |  |  |
| 18:1 w5c |  |  |  |  |  |  | 0.10 |  |  |  |  |  |
| 18:3 w6c (6,9,12) |  | 0.23 |  |  |  |  |  |  |  |  |  |  |
| C _17:1_ *_ω8c_* | 6.41 | 3.97 |  |  |  |  | 6.02 | 4.05 |  |  |  |  |
| C_18:1_ *_ω9c_* | 63.78 | 57.54 |  |  |  |  | 63.81 | 59.2 |  |  |  |  |
| ^#^Sum In Feature 3: 16:1 w7c/16:1 w6c | 13.81 | 17.65 |  |  |  |  | 13.26 | 15.18 | 36.22 | 38.10 | 37.22 | 51.24 |
| ^#^Sum In Feature 18:1 w7c/18:1 w6c |  |  |  |  |  |  |  |  | 23.65 | 22.10 | 6.72 | 9.22 |
| 18:3 w6c (6,9,12) |  | 0.23 |  |  |  |  | 0.27 | 0.27 |  |  |  |  |
| Sum In Feature 6: 19:1 w11c/19:1 w9c | 0.22 |  |  |  |  |  |  |  |  |  |  |  |
| Sum In Feature 7: 19:1 w6c/w7c/19cy |  | 1.01 |  |  |  |  |  |  |  |  |  |  |
| 20:1 w9c |  |  |  |  |  |  | 0.43 |  |  |  |  |  |
| C_17:0_ cyclo |  |  |  |  |  |  |  |  | 3.94 | 1.69 | 13.84 | 2.42 |
| 19:0 cyclo w8c | 0.12 |  |  |  |  |  | 0.12 |  | 0.12 |  |  |  |
| ***Total*** | **90.72** | **80.63** |  |  |  |  | **88.83** | **78.7** | **64.05** | **62.07** | **58.4** | **63.24** |

^#^Summed features represent groups of two or three fatty acids that cannot be separated by GLC with the MIDI system.
