# Supplementary material for: Bacterial diversity and biopotentials of Hamtah glacier cryoconites, Himalaya
Source: Front Microbiol. 2024 May 1;15:1362678. doi: 10.3389/fmicb.2024.1362678 (PMC11094618; doi:10.3389/fmicb.2024.1362678)
Supplement: Supplementary file 8 [file Table_7.docx]

**Supplementary Table 7.** Antibiotic sensitivity test of selected strains of Hamtah glacier cryoconites, Himalaya (3days incubation at 15°C)

| Sr. No | **Antibiotics** | **B2-8** | **B2p7** | **A2-6** | **ECRY-2** | **ECRY-4** | **B2-6** |
| --- | --- | --- | --- | --- | --- | --- | --- |
|  |  | zone of inhibition (mm) | zone of inhibition (mm) | zone of inhibition (mm) | zone of inhibition (mm) | zone of inhibition (mm) | zone of inhibition (mm) |
| 1 | Amikacin AK30 | 19 | 29 | 29 | 24 | 29 | 25 |
| 2 | Ampicillin AMP10 | 0 | 28 | 34 | 29 | 30 | 0 |
| 3 | Azithromycin AT15 | 0 | 27 | 29 | 34 | 29 | 0 |
| 4 | Carbencillin CB100 | 20 | 29 | 28 | 26 | 29 | 22 |
| 5 | Cefaclor CF30 | 0 | 29 | 24 | 26 | 24 | 0 |
| 6 | Cefazolin CZ30 | 0 | 21 | 26 | 15 | 21 | 0 |
| 7 | Cefixime CFM5 | 19 | 19 | 24 | 14 | 19 | 0 |
| 8 | Cefmetazole CMZ30 | 0 | 34 | 28 | 24 | 30 | 0 |
| 9 | Cefoperazone CPZ75 | 20 | 20 | 21 | 8 | 11 | 18 |
| 10 | Cefotaxime CTX30 | 0 | 38 | 34 | 24 | 34 | 24 |
| 11 | Cefoxitin CX30 | 0 | 36 | 34 | 20 | 31 | 0 |
| 12 | Ceftazidine CAZ30 | 10 | 29 | 28 | 19 | 29 | 19 |
| 13 | Ceftriaxone CTR30 | 0 | 28 | 27 | 24 | 24 | 0 |
| 14 | Cefuroxime CXM30 | 0 | 26 | 25 | 19 | 26 | 0 |
| 15 | Cephalothin CH30 | 0 | 17 | 24 | 9 | 14 | 0 |
| 16 | Chloramphenicol C30 | 29 | 28 | 28 | 34 | 30 | 4 |
| 17 | Ciprofloxacin CIP5 | 30 | 34 | 27 | 34 | 29 | 25 |
| 18 | Clidamycin CD2 | 0 | 0 | 0 | 0 | 4 | 0 |
| 19 | Doxycyline Hydrochloride DO30 | 39 | 29 | 29 | 16 | 26 | 24 |
| 20 | Erythromycin E15 | 0 | 14 | 18 | 6 | 7 | 0 |
| 21 | Gatifloxacin GAT5 | 29 | 34 | 34 | 24 | 34 | 29 |
| 22 | Gentamicin GEN10 | 19 | 25 | 24 | 19 | 25 | 21 |
| 23 | Kanamycin K30 | 14 | 26 | 14 | 20 | 25 | 24 |
| 24 | Levofloxacin LE5 | 29 | 28 | 29 | 29 | 36 | 26 |
| 25 | Linezolid LZ30 | 0 | 24 | 19 | 14 | 21 | 0 |
| 26 | Lomefloxacin LO10 | 27 | 22 | 18 | 24 | 28 | 21 |
| 27 | Meropenem MRP10 | 32 | 24 | 31 | 26 | 29 | 28 |
| 28 | Methicillin MET 5 | 0 | 0 | 0 | -6 |  | 0 |
| 29 | Nalidixic Acid NA30 | 30 | 29 | 0 | 34 | 34 | 12 |
| 30 | Netillin NET30 | 24 | 34 | 24 | 24 | 27 | 22 |
| 31 | Nitrofurantoin NIT300 | 0 | 10 | 14 | 9 | 8 | 0 |
| 32 | Norfloxacin NX10 | 16 | 29 | 26 | 21 | 30 | 24 |
| 33 | Ofloxacin OF5 | 21 | 34 | 31 | 20 | 30 | 26 |
| 34 | Oxacillin OX1 | 0 | 0 | 0 | 0 |  | 0 |
| 35 | Penicillin-G P10 | 0 | 23 | 24 | 14 | 19 | 0 |
| 36 | Piperacillin P100 | 0 | 26 | 24 | 17 | 24 | 25 |
| 37 | Polymyxin-B PB300 | 0 | 14 | 16 | 12 | 14 | 14 |
| 38 | Rifampicin R5 | 24 | 24 | 26 | 23 | 14 | 9 |
| 39 | Streptomycin S10 | 25 | 29 | 28 | 28 | 29 | 16 |
| 40 | Teicoplanin TE30 | 4 | 19 | 9 | 4 | 5.5 | 0 |
| 41 | Tetracycline T30 | 37 | 26 | 30 | 24 | 29 | 24 |
| 42 | Ticarcillin T175 | 24 | 28 | 34 | 24 | 29 | 0 |
| 43 | Tobramycin TOB10 | 24 | 24 | 24 | 18 | 20 | 19 |
| 44 | Trimethoprim TR5 | 32 | 25 | 19 | 11 | 19 | 0 |
| 45 | Vancomycin VA30 | 24 | 13 | 13 | 9 | 9 | 0 |
